# Lithiation Gradients and Tortuosity Factors in Thick NMC111-Argyrodite Solid-State Cathodes

**DOI:** 10.1021/acsenergylett.2c02699

**Published:** 2023-02-02

**Authors:** Alyssa M. Stavola, Xiao Sun, Dominick P. Guida, Andrea M. Bruck, Daxian Cao, John S. Okasinski, Andrew C. Chuang, Hongli Zhu, Joshua W. Gallaway

**Affiliations:** † Department of Chemical Engineering, Northeastern University, 360 Huntington Avenue, Boston, Massachusetts02115, United States; ‡ Department of Mechanical and Industrial Engineering, Northeastern University, 360 Huntington Avenue, Boston, Massachusetts02115, United States; § X-ray Science Division, Advanced Photon Source, Argonne National Laboratory, Lemont, Illinois60439, United States

## Abstract

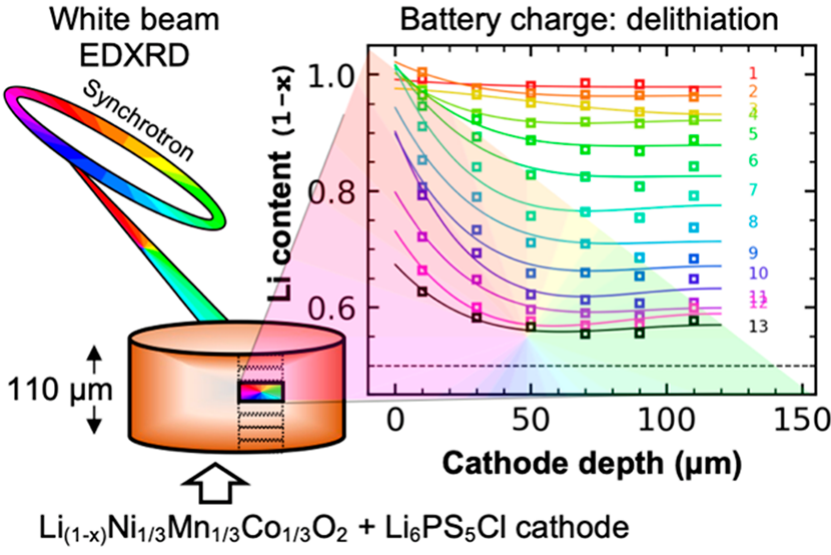

Achieving high energy density in all-solid-state lithium batteries will require the design of thick cathodes, and these will need to operate reversibly under normal use conditions. We use high-energy depth-profiling X-ray diffraction to measure the localized lithium content of Li_1–*x*_Ni_1/3_Mn_1/3_Co_1/3_O_2_ (NMC111) through the thickness of 110 μm thick composite cathodes. The composite cathodes consisted of NMC111 of varying mass loadings mixed with argyrodite solid electrolyte Li_6_PS_5_Cl (LPSC). During cycling at C/10, substantial lithiation gradients developed, and varying the NMC111 loading altered the nature of these gradients. Microstructural analysis and cathode modeling showed this was due to high tortuosities in the cathodes. This was particularly true in the solid electrolyte phase, which experienced a marked increase in tortuosity factor during the initial charge. Our results demonstrate that current distributions are observed in sulfide-based composites and that these will be an important consideration for practical design of all-solid-state batteries.

All-solid-state Li batteries (ASLBs) are under intense research and development due to their potential to replace flammable organic liquid electrolytes with potentially safer solid materials and increase energy density.^1−4^ Several types of Li^+^-conducting solid-state electrolytes (SSEs) are being studied, e.g., sulfides, oxides, phosphates, polymers, and various composites of these.^5−8^ Lithium thiophosphate SSEs so far provide the highest conductivities, often >1 mS/cm.^9−17^ A compelling reason to develop these SSEs is that their high conductivities can transport Li^+^ through possibly tortuous pathways in thick composite cathodes. A recent perspective has stated that to achieve high energy density, the cathode must be the largest component of the battery, suggesting thickness values from 45 to 200 μm.^18^ This is because to balance an energy dense and thin Li metal anode, a composite cathode of comparable capacity will need to be much thicker.^19^ Energy density calculations in Figure S1 demonstrate that achieving a cathode >100 μm will be essential to any practical ASLB.

Most ASLB cathodes presented in the literature remain thin, to focus on phenomena inherent to the materials. However, studies of microstructural effects in cathodes of more practical thickness are also needed for ASLB development.^20^ This Letter concerns high-thickness cathodes prepared with chloride argyrodite SSE, Li_6_PS_5_Cl (LPSC).^21,22^ Specifically, we report the electrochemical nonuniformity within cathodes as a function of the fraction of cathode active material (CAM) in the composite cathode. We measure nonuniformity using energy dispersive X-ray diffraction (EDXRD), which is a synchrotron depth-profiling technique that can be used to obtain spatially resolved diffraction data from within sealed cells.^23−25^ Operando data were obtained under normal use conditions, including a 50 MPa stack compression. The EDXRD setup employed is illustrated in Figure S2, and the size of the gauge volume where data was collected is shown in Figure S3.

The concept of an ASLB with a ∼110 μm thick cathode is shown in Figure 1a, with EDXRD data taken in six 20 μm slices to observe nonuniformity through the cathode thickness. Our finding is that nonuniformity is observed in thick cathodes and that the nature of the large lithiation gradients developed is determined by unfavorable tortuosity of the transport pathways, especially for Li^+^. Additionally, we find that the tortuosity factors during operando battery cycling are different than those calculated for as-assembled cathodes. This evolution is likely due to particle rearrangement occurring during the initial charge.

**Figure 1:**
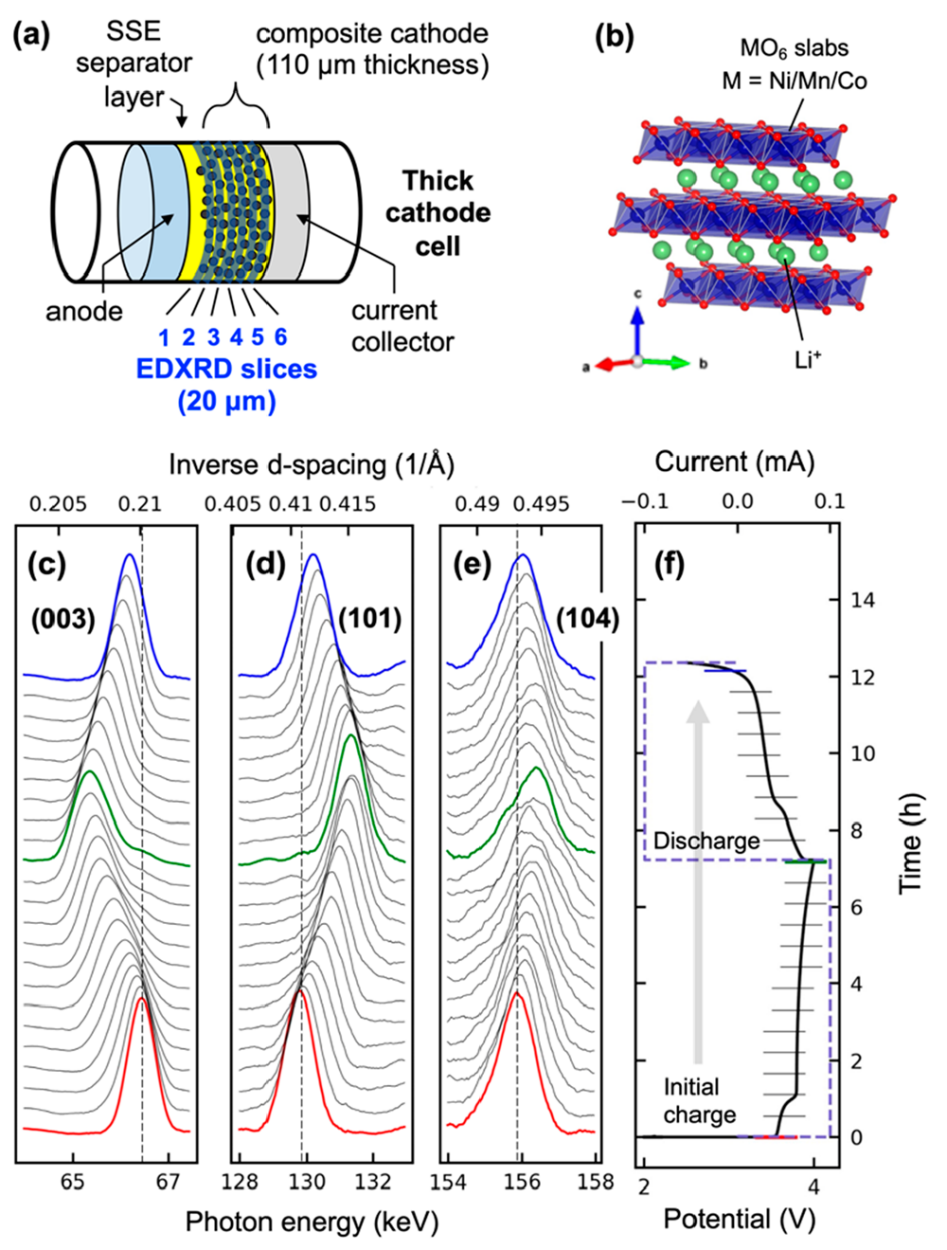
(a) The concept of an ASLB with a thick 110 μm cathode, with EDXRD data taken in six 20 μm slices to observe nonuniformity through the cathode thickness. (b) The structure of NMC111. (c) Operando EDXRD for a cell with a 50 μm thick cathode at C/10. Composition was 70% CAM with a coating.

The CAM of Li-ion batteries is typically a particulate intercalation host, such as NMC111 in this work, which has the electrochemical reaction shown in eq 1.
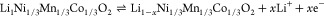
1

During battery charging, the reaction proceeds to the right as the NMC111 is delithiated. The crystal structure of NMC111 is shown in Figure 1b. As Li^+^ is deintercalated, it moves out of the interlayer, and this affects the crystal structure. Since Li^+^ no longer screens the charge of the negative oxygen atoms on the slabs, the slabs are forced apart from each other. If the lattice parameters of the NMC111 are known, the value of *c*/*a* is correlated to the Li content (1 – *x*).^26^ This makes it possible to know the local extent of reaction x in eq 1, which in a nonuniform system will be different than the global extent of reaction calculated from the Ah of capacity charged or discharged. Scanning electron microscopy (SEM) images of NMC111 are shown in Figure S4.

Figure 1c–f shows operando EDXRD data correlated to a charge–discharge cycle of a composite cathode. This particular cell had a thin cathode of ∼50 μm, captured across three EDXRD slices. The CAM in this cell had a coating of Li_0.35_La_0.5_Sr_0.05_TiO_3_ (LLSTO) as protection from the thermodynamic instability between NMC111 and LPSC.^27−31^ SEM images of the NMC111-LPSC are shown in Figure S5. The positions of the (003), (101), and (104) reflections were refined to determine the lattice parameters and therefore the *c*/*a* value. The extent of reaction could then be calculated. Any peak bifurcation or phase separation in the NMC was accounted for using a weighted average of the two peaks corresponding to the two NMC phases, as shown in Figure S6.^32^

Spatial resolution of the (003) is shown in Figure S7. This allowed calculating a local extent of reaction for any particular 20 μm slice of the cathode. In this thin electrode, only small differences in local reaction rate were detected. However, when moving to thicker cathodes, large spatial gradients in lithiation developed.

Spatial distributions of reaction rate in battery materials are important because they inform battery design, which requires knowledge of the uniformity of active material utilization under different use cases. Computational models are one way to determine distributions, but direct measurements are possible in some cases. Operando XRD data used to determine spatially localized state-of-charge (SOC) have been reported for NMC and LFP cathodes;^33−35^ graphite anodes;^36^ and in primary and rechargeable alkaline batteries.^37,38^ Optical methods have also been reported using the SOC-related color change of graphite.^39^

The report by Liu and co-workers illustrates how reaction distributions could affect lifetime health of active material. They used operando XRD computed tomography to observe 3D lithiation gradients in LFP batteries with a liquid electrolyte.^33^ Through the cathode depth, they observed a favored current distribution both at the separator and current collector. This meant there was a peak charging rate for individual cathode particles of 2–3× the nominal rate, with the highest 3× rates being experienced by not only the slices nearest the separator and current collector but also the electrode center, which had the most lagged reaction. This could result in faster CAM degradation at those locations.

One difference between ASLBs and liquid electrolyte batteries is the importance of tortuosity factor. Reported tortuosity values vary widely across the literature, but the tortuosity factor for ionic conductivity in the liquid phase of LCO batteries is on the order of 2–4.^40^ In contrast, composite cathodes based on SSEs have large and highly varying tortuosity factors near the optimal cathode composition. In other words, for ASLBs, CAM loadings are typically optimized from 60 to 75%, and in this regime, tortuosity factor can change greatly with a slight variation in CAM loading.^41,42^

A second important difference is that SSE does not flow to easily wet active material particles. Any structural corruption during cycling such as crack formation in NMC particles will lead to reduced contact area between the active material and electrolyte, potentially reducing accessible capacity. Another difference between ASLBs and liquid electrolyte batteries is that some ASLBs are based on single-ion-conducting SSEs, meaning there are no Li^+^ concentration gradients in the electrolyte. However, this does not necessarily mean the reaction distribution will be curtailed. In composite electrodes using a sulfide-electrolyte SSE, it has been demonstrated that even without Li^+^ concentration gradients, current inhomogeneity through the electrode depth was not suppressed.^39^ In ref (39), current was found to focus at locations nearest to the counter electrode.

In our study, cells were cycled that had a wide variation of CAM mass fraction: 40, 70, and 80%. Cells had 30 mg cathodes, and the current was based on a theoretical C/10 rate (cell areal loadings are reported in Table S1). All experiments were performed at room temperature with an applied stack compression of 50 MPa. To lessen degradation, no carbon additive was used.^43^ Operando EDXRD data was collected by dividing the cathode thickness into slices 20 μm thick. The “0–20 μm” slice was closest to the SSE separator and was termed “slice 1”. Subsequent slices were each 20 μm deeper into the cathode until the final slice, which was next to the current collector foil. The 70% and 80% CAM cells were ∼110 μm thick and had six slices. The 40% CAM cell was ∼155 μm and had eight slices due to the lower density of LPSC.

The initial two cycles for the 80, 70, and 40% CAM cells are shown in Figure 2a–c as operando data correlated to the voltage profiles. The spatially resolved Li content (1 – *x*) showed substantial gradients in lithiation across the cathode thicknesses, particularly during charge 1. Another 70% CAM cell is shown in Figure 2d, which differed only by a coating of LLSTO on the CAM. The Li contents for all cells are also shown as 2D profiles in Figures S8 and S9. For each cell, a local reaction rate was calculated as a transfer current in mA/mm^3^. Voltage-curve-correlated transfer currents are shown in Figure S10.

**Figure 2:**
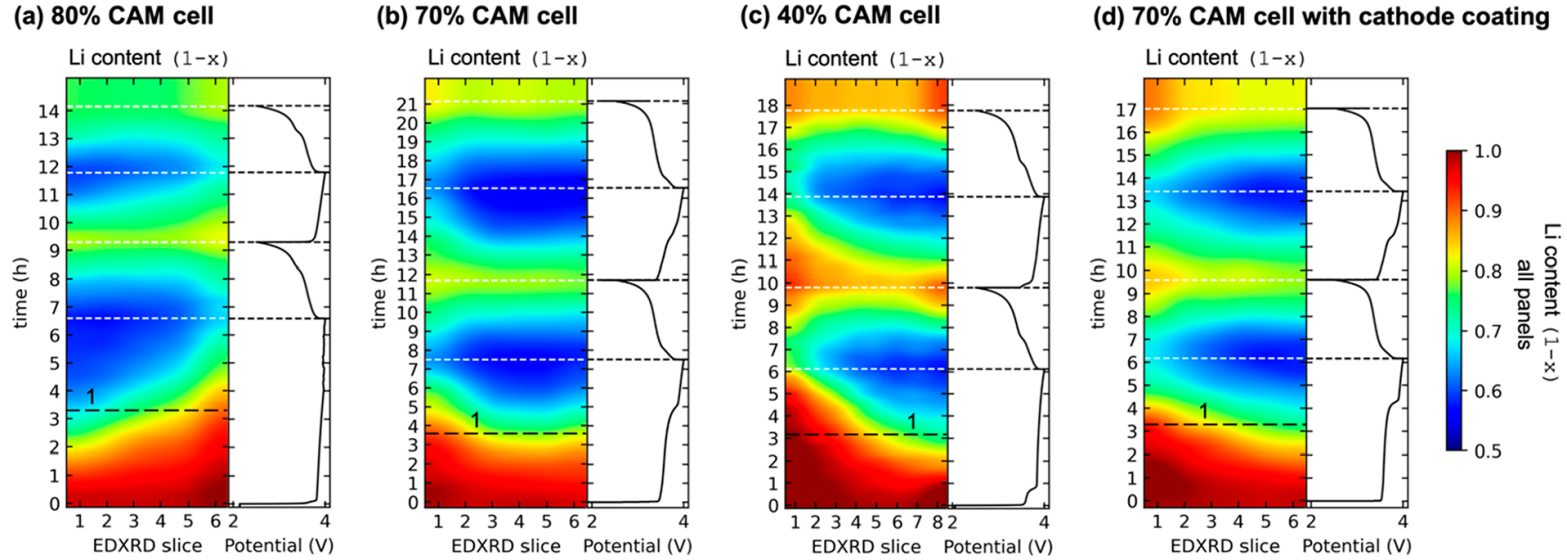
Operando EDXRD data for initial cycling of NMC111-LPSC cathodes as a function of cathode composition. (a) 80% cathode active material (CAM); (b) 70% CAM; (c) 40% CAM; (d) 70% CAM with an LLSTO-coated NMC. For each cell, local Li content (1 – *x*) is shown correlated to the voltage profile.

Some findings were apparent from Figure 2. The first was the magnitude of the lithiation gradients across the cathode thicknesses, which were large by typical Li-ion battery standards. The second was the “flip” in current distribution between the 70% and 80% CAM cells. During charge 1, the 70 and 40% CAM cells showed preferential delithiation near the current collector, while the electrochemical reaction lagged near the separator. For the 80% CAM cell, this trend was reversed: there was strong delithiation at the separator, while the reaction lagged at the current collector. This flip was caused by the effect of tortuosity factor in the cathode. A third finding was that during some times in the 40% CAM cell, the current was reversed. In other words, parts of the cathode continued to charge while the battery was experiencing discharge. A fourth finding was that a CAM coating did not change the nature of the lithiation gradient during charge 1 but did smooth the current distribution subsequently. These findings will be discussed below.

Large gradients are apparent in Figure 3a, which shows the lithiation profile for each cell at the midpoint of charge 1 (marked by note 1 and dashed lines in Figure 2). The 70% CAM cells had the smallest gradients: a maximum Δ*x* of 0.145 without a CAM coating and 0.209 with a coating. These were large by typical Li-ion battery standards, representing 29 and 42% of the total cyclable capacity. Loading of 70% CAM was the optimized composition of the cathodes. Deviations from the optimal loading caused more severe gradients. The 80% CAM cell had a maximum Δ*x* of 0.24 or 48% of capacity. The 40% CAM cell value was 0.338 or 67% of capacity. As described below, these gradients were brought about by the effective electronic and ionic conductivities across the cathodes. Effective conductivities were consequences of the cathode microstructure and thus tortuosity factor.^41,44^

**Figure 3:**
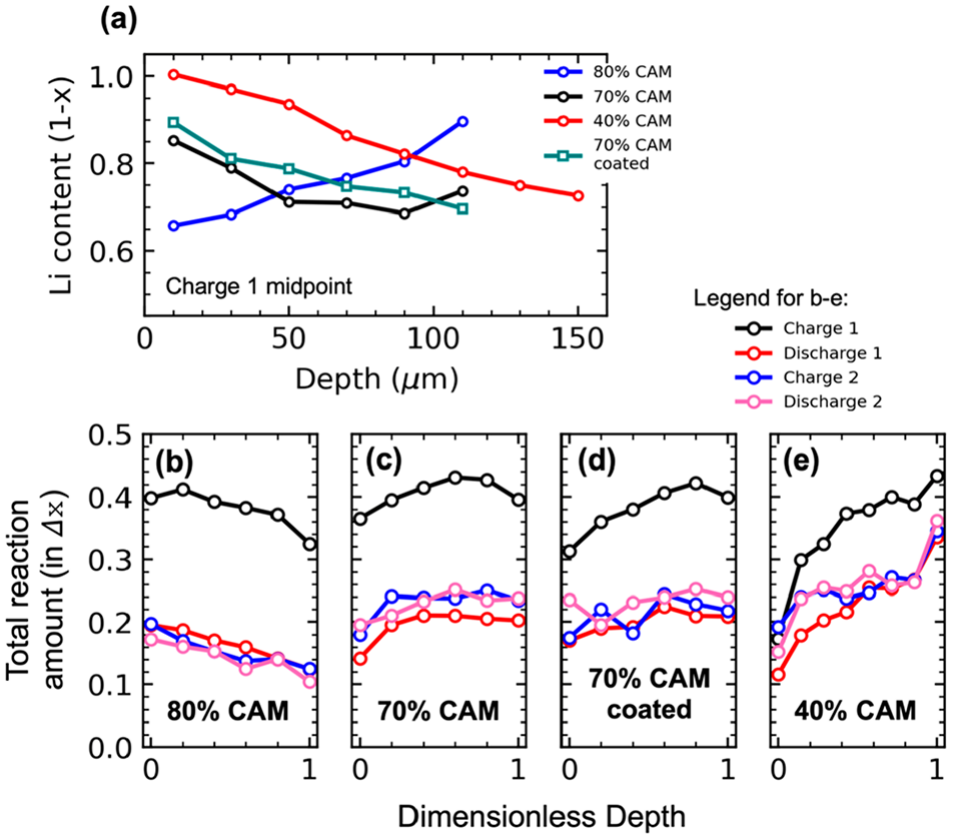
(a) Lithiation profiles for each cell at the midpoint of charge 1. These profiles are marked by note 1 and dashed lines in Figure 2. (b–e) Spatially resolved total reaction amounts in Δ*x* during each cycling stage for (b) 80% CAM; (c) 70% CAM; (d) 70% CAM with a cathode coating; and (e) 40% CAM.

The buildup of these gradients always occurred during charging. In all cells without a CAM coating, gradients were smoothed during discharge, amounting to <0.02 at the end of discharge 1. During charge 2 and discharge 2, coating-free cells again developed significant gradients during charge, and these were smoothed during discharge. In this respect, the 70% CAM cell with a coating was different in that a gradient of 0.07 was maintained even at the end of discharge. This was because the current distribution was more even in this cell after charge 1, and thus, the gradient established initially was maintained to a greater degree.

Figure 3b–e shows the total electrochemical reaction for each cathode slice, for each cycling stage. This is shown as |Δ*x*|, the change in Li content. The first cycle inefficiency observed in the cells is apparent between charge 1 and discharge 1. The largest disparity across a composite cathode was in the 40% CAM cell, which had a relatively efficient reaction at the current collector but a highly lagging and incomplete reaction at the separator. This was because the 40% CAM, which was only 19 vol % CAM, had ineffective percolation pathways for electrons, hindering transport to the separator region.

From the transfer currents in Figure S10, the separator region in the 40% CAM cell showed current reversal at the beginning of each discharge (marked by note 2). This means this region continued to delithiate as the regions closer to the current collector lithiated. During discharge 1, this condition lasted for 1.2 h. This showed the importance of interparticle Li transport, as the lithiation gradient was sufficient to drive Li^+^ from NMC near the separator into particles toward the current collector. Li and co-workers observed current reversal in high-loading NMC811 cathodes during operando XRD experiments.^34^ This effect can be caused by inefficient percolation pathways in ASLBs.

Regarding orientation of the lithiation gradients, Figure 3a shows the 70 and 40% CAM cells experienced a lag in the electrochemical reaction at the separator. However, at 80% CAM, this trend was reversed, with a significant lag at the current collector. Comparison of the 70 and 80% CAM cells reveals the mechanisms that caused this lithiation gradient to flip.

Microstructural analysis of transport across the composite cathode was used to understand the lithiation profiles shown in Figure 2.^42,45,46^ Effective Li^+^ and e^–^ conductivities across the composite cathodes were measured using ion-blocking and electron-blocking cells, as described by Minnmann et al.^42^ These cells, shown in Figure S11, were used to determine each conductivity independently by electrochemical impedance spectroscopy (EIS). The EIS data for cells of different mass% CAM are shown in Figure S12. A T-type transmission line model as described by Siroma et al. was used to extract the ionic resistance *R*_*ion*_ and electronic resistance *R*_*el*_ of each cathode, as shown in Figure S13.^47^ The effective conductivities (*σ*_*i*__,*eff*_) were calculated using the thickness (*L*) and area (*A*) of the cathode and the appropriate resistance *R*_*i*_, where *i* = *ion* or *el*.
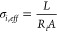
2

The effective ionic conductivity *σ*_*ion*__,*eff*_ and electronic conductivity *σ*_*el*__,*eff*_ of each cathode are shown in Figure 4a as a function of the mass% CAM in the cathode.^47^ As CAM approached 100%, *σ*_*el*__,*eff*_ approached the e^–^ conductivity of NMC111. As CAM approached 0%, *σ*_*ion*__,*eff*_ approached the Li^+^ conductivity of LPSC. Effective conductivities varied over orders of magnitude as the mass% CAM was varied. It should be noted that these conductivities characterized the electrode material in its initial, i.e., fully lithiated, state. The conductivity of LPSC is constant, but NMC111 has a variable e^–^ conductivity that increases as (1 – *x*) decreases, and thus, *σ*_*el*__,*eff*_ evolved during initial charging.

**Figure 4:**
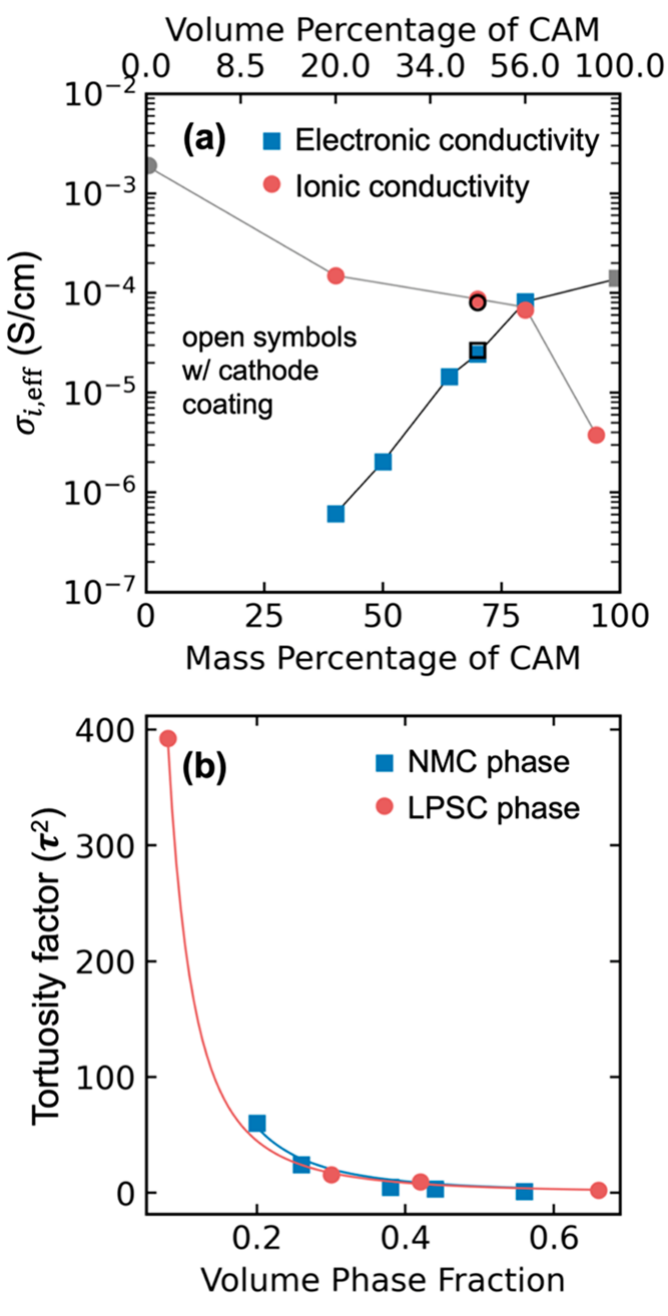
(a) Effective electronic conductivity *σ*_*el*__,*eff*_ and ionic conductivity *σ*_*ion*__,*eff*_ of composite cathodes as a function of mass% CAM. Open markers at 70 mass% are coated CAM data. (b) Fitted tortuosity factor of each phase as a function of phase fraction.

Tortuosity factor τ^2^ was calculated using the bulk conductivity (σ_*i*,*bulk*_), the effective partial conductivity (σ_*i*,*eff*_), and the volume fraction (ϵ_*i*_) of the material. Volume fraction was calculated assuming 14% of the cathode is void.^7,41,42,48,49^
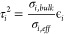
3

The relation between tortuosity factor and volume fraction is often given in the power-law form of eq 4.
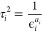
4

For a value of *a*_*i*_ = 0.5, this is termed the Bruggeman correlation, which is frequently used in battery transport models.^50^ Fitting in Figure 4b determined *a*_*LPSC*_ = 2.36 and *a*_*NMC*_ = 2.51 (here *i* is the name of the relevant phase). These values indicated high tortuosity factors in both phases: τ_*LPSC*_^2^ = 7.78 and τ_*NMC*_^2^ = 7.86 for 70% CAM; τ_*LPSC*_^2^ = 17.24 and τ_*NMC*_^2^ = 4.29 for 80% CAM. This revealed the LPSC phase was near a tipping point at 70% CAM, with τ_*LPSC*_^2^ more than doubling at 80% CAM. However, it was desired to understand the evolving tortuosity in an operating cell. To quantify this, a cathode model was developed and correlated to the operando EDXRD data in Figure 2a,b, with *a*_*i*_ as fitting parameters.

COMSOL Multiphysics 6.0 was used to model the charge of composite cathodes, with results shown in Figure 5a–c and Figure 5d–f for 70 and 80% CAM. This model included an SOC-dependent exchange current density *i*_*o*_ as well as increasing e^–^ and Li^+^ conductivities for NMC as Li content fell.^51,52^ The model domain was two rows of circular NMC particles, each in contact with a single-ion-conducting LPSC channel, shown in Figure S14. Conductivities of the NMC particles and LPSC channels were modified by eq 3 to simulate the effects of phase fraction and tortuosity factor on transport across the composite cathode. COMSOL model details are given in Tables S2–S4. Details of the EIS model used for Figure 4a results are given in Tables S5–S8.

**Figure 5:**
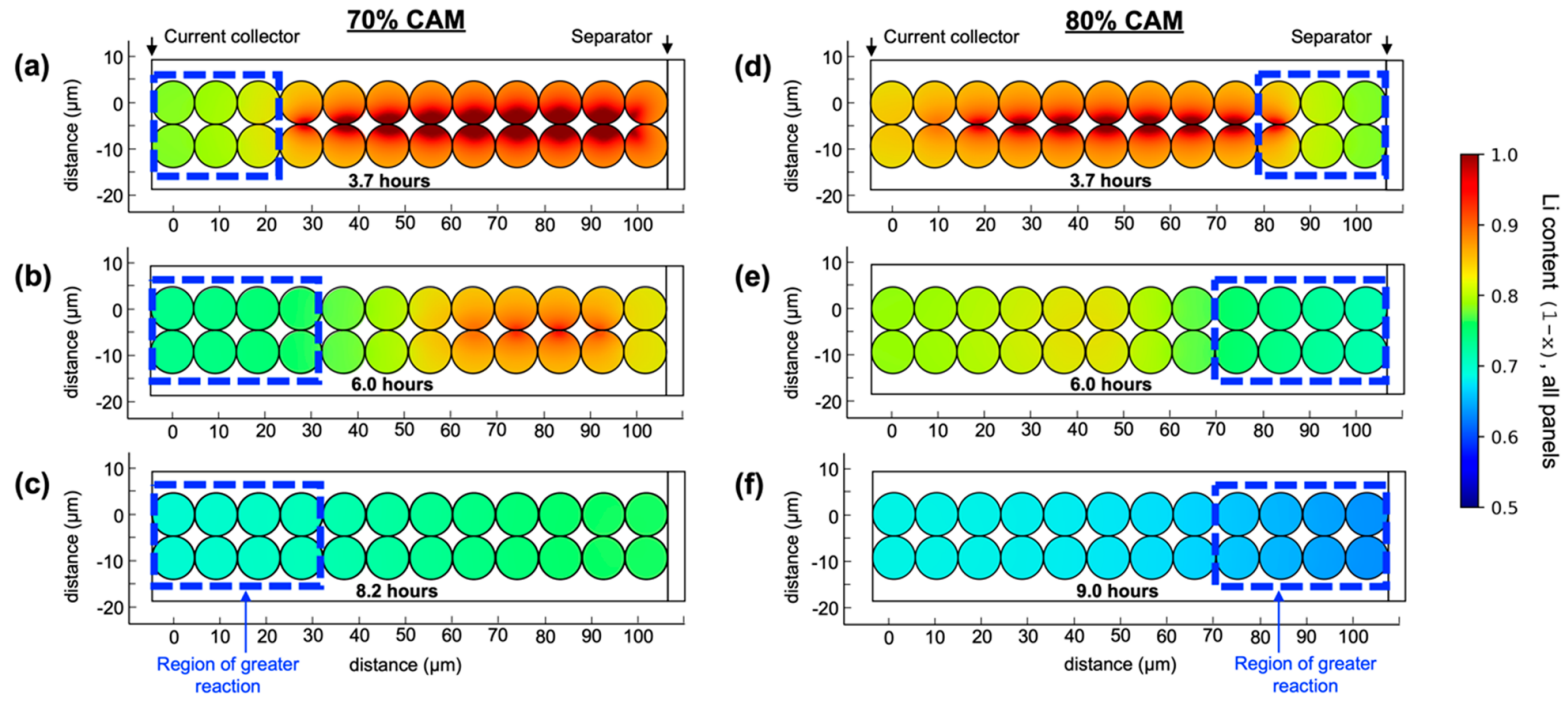
COMSOL simulations during initial charge showing local Li content (1 – *x*) as a function of cathode depth: (a–c) 70% CAM cell with greater reaction at the current collector. (d–f) 80% CAM cell with greater reaction at the separator.

Fitting parameters *a*_*LPSC*_ and *a*_*NMC*_ were used to match the model results to those in Figure 2a,b. Good agreement with the 70–80 flip and the Li content values was obtained at *a*_*LPSC*_ = 2.7 and *a*_*NMC*_ = 2.0. In comparison with the symmetric cell results, this was higher for the LPSC phase and lower for the NMC phase. This revealed that the tortuosity factor evolved in composite cathodes during cycling under compression. Because NMC shrinks during delithiation, rearrangement of particle contacts is expected. Shi and co-workers have reported that good ionic transport can be enabled by increasing the size ratio of CAM particles to that of the SSE.^53^ This suggests that during shrinkage of the CAM during initial charge, this ratio changes, and ionic conductivity is negatively impacted. While the NMC values were near those derived from the EIS experiments, tortuosity factors in LPSC were higher: τ_*LPSC*_^2^ = 10.4 for 70% CAM and τ_*LPSC*_^2^ = 25.8 for 80%. These were extraordinary values but were in general agreement with other works considering tortuosity in similar systems.^41,42^ Bielefeld and co-workers have demonstrated that tortuosity effects in ASLBs are determined by complex point contacts between particles and can be nonintuitive.^41^ Greater understanding of tortuosity in ASLBs is needed, with an emphasis on operando measurement of the evolving tortuosity factors as the cathode cycles.

Tortuosity factor was the primary variable affecting the lithiation gradients and 70–80 flip. To assess the contribution of kinetics, *i*_0_ was varied widely in the computational model, producing only small changes in the lithiation gradient results in Figure 5. This confirmed the system was not under kinetic limitation, in agreement with findings by Naik and co-workers that predict this system would be transport-limited.^54^ To ensure that the choice of *i*_*o*_ function did not play an important role, the modeling was repeated using the SOC-dependent *i*_*o*_ of Liu et al. (Figure S15), producing similar results.^55^ The kinetic expression had little impact on results, while the tortuosity factors were of critical importance to matching experiment and model.

All tortuosity factors calculated in this work are listed in Table S9.

Peak bifurcation has been frequently reported in NMC diffraction patterns and was observed in the current work. This peak bifurcation has been described variously as a bimodal composition during delithiation;^32^ as a particle size effect;^51^ and as a consequence of conductive pathways.^52^ While all of the cells tested in this work displayed some amount of peak bifurcation, it was markedly different when the NMC was bare versus coated. This is shown in Figure S16a for the 70% CAM-coated case and in Figure S16b for uncoated 70% CAM.

Peak bifurcation occurred in the coated case, most evident at 4.0 h. However, the left and right peaks were never fully separated in this cell. Comparing the uncoated case, peak bifurcation was more pronounced, with two clear peak maxima at 4.7 h. Here, note the bifurcation difference between the red and purple data, which reveal that at 4.7 h the reaction was more progressed at the current collector than at the separator. To illustrate this difference in peak bifurcation, the voltage profiles in Figure 6 are shown correlated to the left and right peak energies (in keV) and the peak heights (marker sizes). To simplify the plot, only EDXRD slices 1, 3, and 6 are shown; the color-shaded regions show the weighted standard deviations of the right peak (orange) and left peak (blue) including all slices.

**Figure 6:**
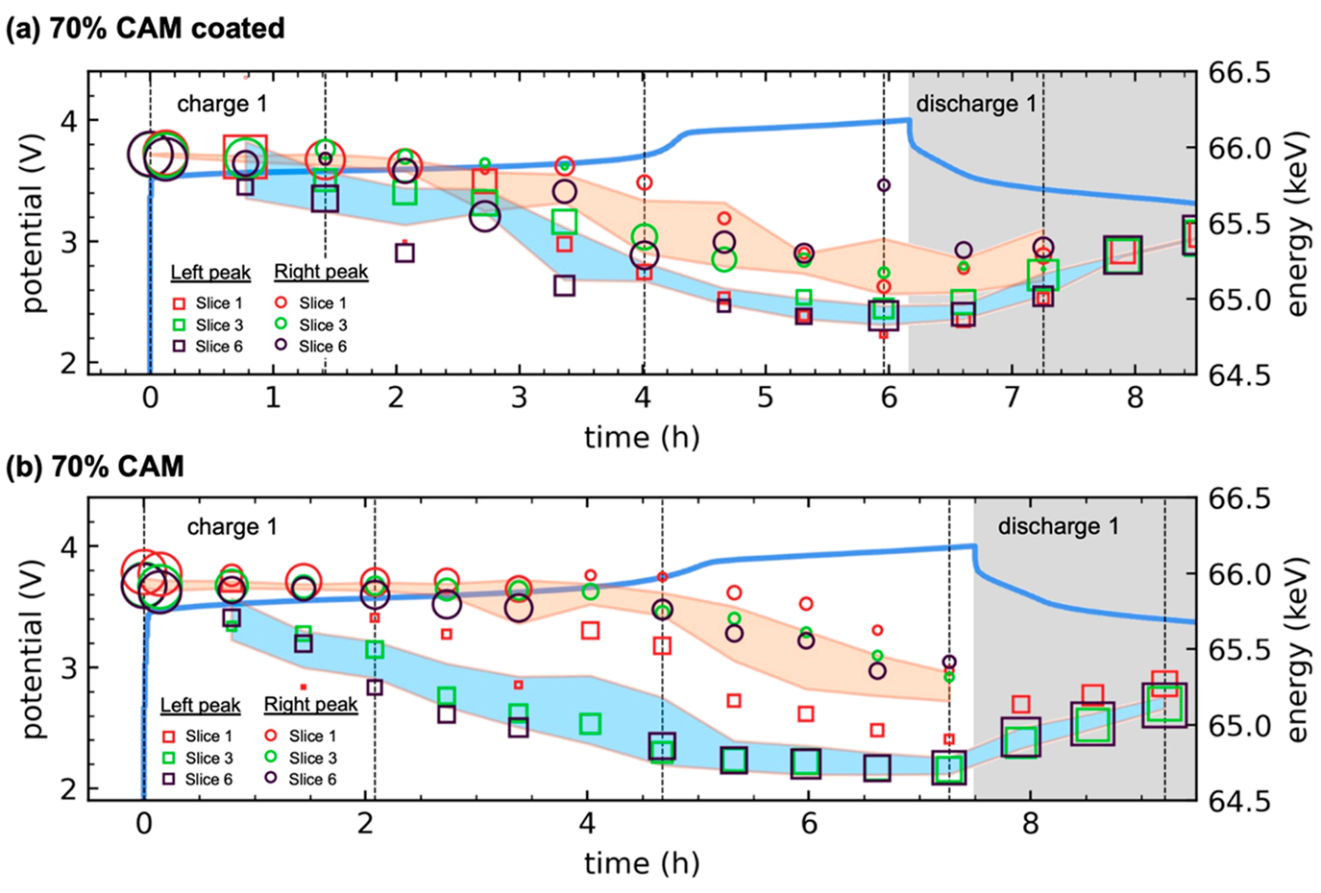
Peak bifurcation of the NMC111 (003) reflection during charge 1, as a function of time and depth in the cathode. (a) 70% CAM with a coating. (b) Uncoated 70% CAM. Photon energies of the bifurcated peaks are shown, with the right and left peaks given by circles and squares. Peak magnitude is indicated by the marker size. Color-shaded regions show the weighted standard deviations of the photon energies. Dashed lines correspond to the times of the data in Figure S16. The conclusion is that peak bifurcation was more significant in the uncoated cell.

For the coated 70% CAM (Figure 6a), the orange and blue regions closely tracked each other, meaning peak bifurcation was weak. For the uncoated 70% CAM (Figure 6b), the regions were well-separated, meaning there was meaningful peak separation throughout charge 1. This is because the uncoated CAM likely had a higher amount of decomposition products on the NMC surface, caused by the thermodynamic instability between LPSC and NMC. Surface films have been reported to contribute to peak bifurcation in layered transition metal oxides.^56^ This change in the bifurcation behavior agrees with the hypothesis in ref (51) that a reduced value of *i*_*o*_ would result in increased bifurcation. The coated NMC likely had fewer of these decomposition products, a higher *i*_*o*_, and therefore less bifurcation.^21^

For the uncoated 70% CAM, comparison of Figures 6b and 2b reveals that bifurcation was strongest at a local Li content of 0.7–0.75, corresponding to the green shades in Figure 2b. This was where the left peak overtook the right peak in size. For EDXRD slices 3 and 6, Figure 6b shows this occurred from 4.0 to 5.5 h. For EDXRD slice 1, which was near the separator and lagged, this occurred from 5.5 to 6.5 h.

Results in ref (51) suggested that bifurcation may be provoked by high relative current. However, Figure S10a shows that EDXRD slice 1 of the 80% CAM cell experienced the highest transfer current of any NMC material in this study, from 2.5 to 3.5 h. This also coincided with the appearance of the left NMC peak, and Figure S17 shows that this location showed relatively low peak bifurcation. We attribute this to the high amount of current focused on this region, which caused both the left and right peaks to shift simultaneously. Thus, this suggests high current can actually mask bifurcation locally, making the two peaks difficult to resolve. By comparison, EDXRD slice 6, which was highly lagged in this cell, showed a large bifurcation, which remained through charge 1 and well into discharge 1.

In this work, we have demonstrated spatially resolved current distributions in NMC111-argyrodite composite cathodes. These result in lithiation gradients, meaning local SOC of the CAM does not match the average SOC calculated from the capacity withdrawn and the CAM mass within the cell. In composite ASLB cathodes of commercially relevant thickness, lithiation gradients will be an important consideration.

1. Like traditional, liquid electrolyte cathodes, the current distribution can favor either side of the electrode: the separator or current collector side, depending on conditions. Unlike liquid electrolyte cells, this system has a high tortuosity factor, with large changes in effective conductivity provoked by relatively small changes in loading.
2. Tortuosity factors were large and found to change during initial charging, with the solid electrolyte tortuosity factor increasing significantly.
3. NMC peak bifurcation was observed at a relatively low rate of C/10. Bifurcation was also spatially resolved and occurred when local Li content was 0.7–0.75. If current was highly focused on a cathode region during this time, bifurcation was masked as both peaks shifted together.

## Supporting Information Available

Experimental details; cathode model equations; energy density calculations; EDXRD setup; microscopy images of electrode materials; bifurcated peak fitting; lithiation profiles in 2D; volumetric transfer current data; EIS data and transmission line model; COMSOL domains; EDXRD peak bifurcation data; operando peak bifurcation plots for 80% CAM; supplemental references (PDF)
