# Supplementary material for: Lithiation Gradients and Tortuosity Factors in Thick NMC111-Argyrodite Solid-State Cathodes
Source: ACS Energy Lett. 2023 Feb 2;8(2):1273–80. doi: 10.1021/acsenergylett.2c02699 (PMC10629242; doi:10.1021/acsenergylett.2c02699)
Supplement: Supplementary file 1 — nz2c02699_si_001.pdf [file nz2c02699_si_001.pdf]

Supporting Information

Lithiation Gradients And Tortuosity Factors In Thick NMC111-Argyrodite Solid-State Cathodes

Alyssa M. Stavola [1], Xiao Sun [2], Dominick P. Guida [1], Andrea M. Bruck [1], Daxian Cao [2], John S. Okasinski [3], Andrew C. Chuang [3], Hongli Zhu *, [2], and Joshua W. Gallaway *, [1]

1. Department of Chemical Engineering, Northeastern University, 360 Huntington Avenue, Boston, MA 02115, USA.

2. Department of Mechanical and Industrial Engineering, Northeastern University, 360 Huntington Avenue, Boston, MA 02115, USA.

3. X-ray Science Division, Advanced Photon Source, Argonne National Laboratory, Lemont, Illinois 60439, USA.

1. Experimental Section

Materials

The cathode active material (CAM) was NMC111, purchased from MSE Supplies, Tucson, AZ. The solid-state electrolyte (SSE) precursors P_2S_5 (99%), Li_2S (99.98%), and LiCl (99%) were purchased from Sigma-Aldrich. For the cathodes with coated CAM, $Li_{0.35}La_{0.5}Sr_{0.05}TiO_3$ (LLSTO) was prepared and coated with a wet chemical approach on NMC111 as per a previous publication.[1] This coating of LLSTO was found to be amorphous and 15-20 nm thick by XRD and TEM.

LPSC preparation

Argyrodite Li_6PS_5Cl (LPSC) was synthesized by mechanical ball milling and then annealing treatment.[1] A stoichiometric mixture of P_2S_5, Li_2S, and LiCl was milled in an stainless steel jar (50 mL) with zirconia balls (10 mm in diameter) and zirconia balls (5 mm in diameter) for 10 hours at 500 rpm. Then the as-mixed precursors were sealed in a glass tube and annealed in a quartz tube furnace at 550 ºC for 6h.

Fabrication of ASLBs

To prepare the cathode, NMC111 was manually mixed with Li_6PS_5Cl in a mortar with the ratio specified in the manuscript. Two hundred milligrams of Li_6PS_5Cl were first pressed into a pellet with a diameter of 12.6 mm under 300 MPa. After that, 30 mg of as-prepared cathode was casted onto the pellet and pressed at 100 MPa. A piece of In−Li was added as the anode to the opposite side by pressing at 100 MPa. Cu and Al foils were selected as the current collectors on the anode and cathode sides respectively. An extra pressure of 50 MPa was applied during cycling and operando testing, using a custom-built compression jig. Areal loading of NMC was as indicated in **Table S1**, as a function of mass% of cathode active material (CAM). Some 10 mg cathodes for 70% CAM were also made with a similar procedure.

Table S1. Areal loading of NMC.

**30 mg cathode**	
40% CAM	9.5 mg/cm^2
70% CAM	16.6 mg/cm^2
80% CAM	19.0 mg/cm^2

10 mg cathode	
70% CAM	5.5 mg/cm^2

Operando EDXRD Cycling and Analysis

Energy dispersive X-ray diffraction (EDXRD) experiments were conducted at Argonne National Laboratory at beamline 6BM-A at the Advanced Photon Source (APS).[2] A germanium detector at a fixed angle of 2θ = 2.26° was used to measure the diffracted beam. The white beam had an energy range of 20–200 keV. The lower energy (<50 keV) photons were removed using a metal filter. The gauge volume had dimensions of 2 mm by ~5.6 mm by 0.02 mm defined by collimation slits of d_i = 0.02 mm and d_s = 0.2 mm (see **Fig S4**). The incident beam penetrated the cell containment, allowing characterization within the pressurized cell container during cycling. A LaB_6 standard was used to calibrate the detector channel number to inverse d-spacing, and a Savitzky–Golay filter was used to smooth the raw diffraction data.

The cells tested at APS were cycled with a constant current of C/10 between 2.5 and 4.0 V (vs Li−In). Here 1C means 160 mA/g based on the weight of active material in the cathode. The 30 mg composite cathodes were measured to be ~110 μm thick in the 70% and 80% CAM cases. For 40% CAM the thickness was ~155 μm. The EDXRD gauge volume was set to separate the cathode into slices of 20 μm height vertical. The scan time was 10 s per gauge volume for the 70% CAM cells, and 30 s per gauge volume for the 80% and 40% CAM cells.

The lattice parameters of the cathode material (NMC111) are calculated using least squares fit of three of the peaks (003, 101, 104). These peaks are chosen based on the intensity of the EDXRD pattern (60–150 keV being the most visible energy range), as a large signal to noise ratio is necessary to calculate parameters. The (10-2) peak, while within this viable range, showed interference with the Al foil current collector, and was therefore excluded from the fit. **Eq S1** was used to relate d-spacing to lattice parameters (a and c) and miller indices (hkl) for a hexagonal crystal structure.[3]

$$\frac{1}{d^2} = \frac{h^2 + k^2}{a^2} + \frac{l^2}{c^2} \qquad \text{[S1]}$$

Where two 003 peaks were observed a double Gaussian fit and a weighted average was used to calculate the peak maxima. The ratio of the two lattice parameters (c/a) was correlated to the amount of lithium in the NMC111, using a previously published study.[4] This study found that for $Li_{(1-x)}Ni_{1/3}Mn_{1/3}Co_{1/3}O_2$ (from 0<x<0.5), $x = (^c/_a - 4.9722)/0.3552$.

Electrochemical Impedance Spectroscopy

Electrochemical impedance spectroscopy (EIS) was used to characterize the electronic and ionic conductivities across the thickness of composite cathodes. An ion-blocking cell (**Fig S11a**) was used to determine electronic conductivity. These cells had a 30 mg composite cathode pressed into pellet with 50 MPa pressure. The two side current collectors were stainless steel. An electron-blocking cell (**Fig S11b**) was used to determine ionic conductivity. In these cells, 150 mg of SSE was pressed with 150 MPa pressure, then the 30 mg cathode was added with 150 MPa pressure. Then another 150 mg SSE was added with 150 MPa pressure. Two pieces of In-Li were added on the sides of the previous pellet. Stainless steel was used as current collector. The pressure was 50 MPa under all EIS tests. EIS was performed from 1 MHz to 0.01 Hz at a 10 mV amplitude with 6 points per decade using a BioLogic SP-150 potentiostat.

Tortuosity Factors

This work concerns tortuosity factors in composite ASLB cathodes. *Tortuosity* τ is often defined as the shortest transport pathway through a composite microstructure divided by the distance between the start and end points, or $\tau = \Delta l/\Delta x$.[5] The *tortuosity factor* τ^2 is defined by **Eq 3** in the manuscript. These concepts are related but are not the same. In practice the tortuosity can be known when the microstructure is well-defined and understood in detail. The tortuosity factor is empirical, accessible through experiment, and relates a bulk property to an effective or measured property. Importantly, the empirical tortuosity factor accounts not only for the length of conduction paths, but also their width, including cross-sectional areas and point contacts. Tortuosity factors are used extensively in battery models where the microstructural details of composite electrodes are homogenized. It should be noted that the terminology surrounding tortuosity varies in the literature. In this work we use the definitions of Bielefeld et al.[5] In Landesfeind et al. they define the path-length tortuosity, geometric tortuosity, and the effective tortuosity (τ).[6] This last is equivalent in definition to the tortuosity factor discussed above. A thorough review of these differing concepts is outside the scope of this work.

Cathode Modeling

COMSOL Multiphysics 6.0 was used to model the charge of an NMC cathode impregnated with LPSC solid electrolyte. The built-in Battery Design and Transport of Diluted Species modules were used to assemble the governing equations for the system, along with parameters both experimentally determined and reported in relevant literature. The model used the Time Dependent with Initialization study to compute Li content across the cathode during a 10-hour charge. Current density type was set to “Secondary” for initialization to account for electrode kinetics. The time dependent solution was obtained using the Backward Differentiation Formula (BDF) solver with default settings.

A heterogeneous model geometry was built assuming 24 uniform NMC particles with a diameter of 9.3 microns, assembled into two rows to simulate a cathode thickness of 110 microns. The two rows of NMC particles were flanked with ion-conducting channels of LPSC, in contact with bulk electrolyte at one end of the cathode. The two NMC particles on the opposite end of the bulk electrolyte domain were in good electronic contact with the cathode current collector. This geometry was used to represent a 70% CAM and 80% CAM cathode by modifying the total capacity and effective electronic conductivity of the NMC domain, as well as the effective ionic conductivity of the LPSC channels based on the volume fractions calculated for the 70% CAM and 80% CAM cases. The model was used to simulate 10 hours of charge at C/10, based on the total capacity defined by **Eq S2**.

$$I_{total} = \frac{V_{NMC} \cdot \rho_{NMC} \cdot m_{NMC} \cdot SC_{NMC}}{10\ hrs.} \quad [S2]$$

The NMC particle domain was taken to be one contiguous phase of NMC under the assumption that all particles were in sufficient contact that Li ions could freely diffuse across the entire domain. Therefore, COMSOL’s Transport of Dilute Species module was used to calculate the flux of Li ions within the NMC domain in accordance with **Eq S3**.

$$J_{Li} = -D_{NMC} \nabla C_{Li} \quad [S3]$$

Given the dependence of Li diffusivity in NMC on the local concentration of Li within the NMC lattice, the diffusivity of Li within $\mathrm{Li_xNi_{1/3}Mn_{1/3}Co_{1/3}O_2}$ was defined by a piecewise function shown by **Eq S4**, based on the findings of Sharma et al.[7]

$$D_{NMC} = 5 \cdot 10^{\frac{4(1-x)}{0.25}-17}\,\frac{m^2}{s} \quad where\ x\ \in\ [1,0.75]$$
$$D_{NMC} = 5 \cdot 10^{-13}\,\frac{m^2}{s} \quad where\ x\ <\ 0.75 \qquad [S4]$$

In addition to the dependence of diffusivity on Li concentration, the bulk electronic conductivity of the NMC domain was also taken to be a function of Li concentration. Similarly based on the findings of Sharma et al., the bulk electronic conductivity of NMC was defined by **Eq S5**.[7]

$$\sigma_{NMC,bulk} = 10^{\frac{2(1.05-x)}{0.8}-4}\,\frac{S}{cm} \qquad [S5]$$

This bulk conductivity was then converted to the effective electronic conductivity of the NMC domain by taking the phase fraction and tortuosity factor of the phase into consideration. The Bruggeman factors for the NMC and LPSC phases a_{NMC} and a_{LPSC} were tuned to match EDXRD results, through the effective electronic and ionic conductivities. The effective electronic conductivity of the NMC domain was defined by **Eq S6**.

$$\sigma_{NMC,eff} = \sigma_{NMC,bulk} \cdot \varepsilon_{NMC}^{1+a_{NMC}} \qquad [S6]$$

The effective ionic conductivity of the LPSC channels, was given by **Eq S7**.

$$\sigma_{LPSC,eff} = \sigma_{LPSC,bulk} \cdot (1 - \varepsilon_{void} - \varepsilon_{NMC})^{1+a_{LPSC}} \qquad [S7]$$

The values determined through matching the model to EDXRD results in this study are given in **Table S2**.

Table S2. Tuned values for a determined from EDXRD results

a_{NMC}	2.0
a_{LPSC}	2.7

COMSOL's Battery Design module was used to assemble the necessary equations to solve the electronic and ionic current flow across the cathode. Firstly, charge conservation and Ohm's law were used to obtain **Eq S8** and **Eq S9**, where β was used to represent a given phase, namely NMC and LPSC.

$$\nabla \cdot i_\beta = 0 \qquad [S8]$$

$$i_\beta = -\sigma_\beta \nabla \phi_\beta \qquad [S9]$$

The electrode surface reaction was then defined by **Eq S10**, with the local current density of each finite element being assumed to be governed by Butler-Volmer kinetics, shown in **Eq S11**.

$$\boldsymbol{n} \cdot \boldsymbol{i}_{LPSC} = -\boldsymbol{n} \cdot \boldsymbol{i}_{NMC} = i_{total} \qquad [S10]$$

$$i_{loc} = i_0\left(exp\left(\frac{\alpha_a F\eta}{RT}\right) - exp\left(-\frac{\alpha_c F\eta}{RT}\right)\right) \qquad [S11]$$

The expression used to define the exchange current density varies widely in relevant literature sources, with a brief overview of definitions summarized in **Table S3**. Of these sources, both constant and Li concentration-dependent exchange current densities have been used in modeling various Li-ion cathode materials.

Table S3: Overview of expressions used to define exchange current density in relevant literature. Subscripts of SSE denote a simplifying assumption that the expression $\left(C_{electroyte}/C_{elect.,ref}\right)$ can be taken as unity when using a solid-state electrolyte.

Equation for Exchange Current Density	Reference (system)
$i_{0,SSE} = 96485\frac{C}{mol}\cdot 10^{-11}\frac{m}{s}\left(49000\frac{mol}{m^3} - \boldsymbol{C_{Li}}\right)^{0.5}\boldsymbol{C_{Li}^{0.5}}$	7 (NMC532, NMC811)
$i_{0,SSE} = 96485\frac{C}{mol}\cdot 1.183\cdot 10^{-11}\frac{m}{s}(48207 - \boldsymbol{C_{Li}})^{0.5}\boldsymbol{C_{Li}^{0.5}}$	8 (NMC111)
$i_{0,SSE} = 96485\frac{C}{mol}\cdot 5\cdot 10^{-11}\frac{m}{s}\left(50290\frac{mol}{m^3} - \boldsymbol{C_{Li}}\right)^{0.5}\boldsymbol{C_{Li}^{0.5}}$	9 (NMC,NCA)
$i_0 = 96485\frac{C}{mol}\cdot 1.6\cdot 10^{-4}\frac{m}{s}\left(36224\frac{mol}{m^3} - \boldsymbol{C_{Li}}\right)^{0.5}\boldsymbol{C_{Li}^{0.5}}$	10 (NMC111)
$i_{0,SSE} = 96485\frac{C}{mol}\cdot 2\cdot 10^{-11}\frac{m}{s}\left(49000\frac{mol}{m^3} - \boldsymbol{C_{Li}}\right)^{0.5}\boldsymbol{C_{Li}^{0.5}}$	11 (NMC622)
$i_0 = \frac{V_{NMC}\cdot\rho_{NMC}\cdot m_{NMC}\cdot SC_{NMC}}{SA_{NMC}}\cdot 10^{\frac{1-x}{0.075}-2}\frac{A}{m^2} \quad where\ x\ \in\ [1,0.9]$ $i_0 = \frac{V_{NMC}\cdot\rho_{NMC}\cdot m_{NMC}\cdot SC_{NMC}}{SA_{NMC}}\cdot 10^{\frac{0.1}{0.075}+\frac{0.9-x}{0.35}-2}\frac{A}{m^2} \quad where\ x\ <\ 0.9$	12 (NMC111)
$i_0 = 1.26\frac{A}{m^2}$	13 (NMC622)

*The piecewise function is based on an estimation in ref 12 (Figure 5c therein) to compute the exchange current normalized to cathode capacity. The capacity of the NMC domain and the surface area of that domain in contact with the LPSC domain are used to obtain the exchange current density.

It was determined that the expression used to define the exchange current density had only minor impact on the results of the model when using expressions similar to that seen in **Table S3**. Therefore, the exchange current density for the model shown in the main text was defined by a piecewise function shown in **Eq S12**, based on the findings of Park et al.[12]

$$i_0 = \frac{C_{NMC}}{SA_{NMC}} \cdot 10^{\frac{1-x}{0.075}-2} \frac{A}{m^2} \quad where\ x \in [1, 0.9]$$

$$i_0 = \frac{C_{NMC}}{SA_{NMC}} \cdot 10^{\frac{0.1}{0.075}+\frac{0.9-x}{0.35}-2} \frac{A}{m^2} \quad where\ x < 0.9 \qquad [S12]$$

Boundary conditions for the system were satisfied by setting the counter electrode to have an arbitrary potential of $0\ V$, and constraining the cathode current collector using **Eq S13**.

$$I_{total} = -\int_{\delta\Omega} \boldsymbol{i}_{NMC} \cdot \boldsymbol{n} dS \qquad [S13]$$

This system of equations was then solved simultaneously by COMSOL for each finite element of the defined geometry in conjunction with the parameters defined in **Table S4**.

Table S4: Brief description and value of the symbols involved in the model. Parameters with a dashed value entry are not explicitly defined and are computed by COMSOL.

Symbol	Description	Value	Ref.
$\sigma_{NMC,bulk}$	Bulk electronic conductivity of NMC	Eq S5	7
$\sigma_{SSE,bulk}$	Bulk ionic conductivity of LPSC	$2 \cdot 10^{-3} \frac{S}{cm}$	
$\sigma_{NMC,eff}$	Effective electronic conductivity of NMC	Eq S6	
$\sigma_{SSE,eff}$	Effective ionic conductivity of LPSC	Eq S7	
$\varepsilon_{NMC,70CAM}$	Volume fraction of NMC, 70CAM cathode	0.44	
$\varepsilon_{NMC,80CAM}$	Volume fraction of NMC, 80CAM cathode	0.56	
ε_{void}	Volume fraction of void	0.14	5
D_{NMC}	Li diffusivity in NMC	Eq S4	7
i_0	Exchange current density	Eq S12	12 and others (see text)
i_{NMC}	Electronic current density	-	
i_{LPSC}	Ionic current density	-	
i_{loc}	Local current density	-	
i_{total}	Total current density	-	
I_{total}	Total charge current	Eq S2	

ϕ_{NMC}	Electronic potential	-	
ϕ_{LPSC}	Ionic potential	-	
C_{Li}	Li content in NMC	-	
$C_{s,max}$	Maximum Li content in NMC	$4.9 \cdot 10^4 \frac{mol}{m^3}$	7
α_a	Anodic transfer coefficient	0.5	7
α_c	Cathodic transfer coefficient	0.5	7
η	Overpotential	-	
J_{Li}	Li ion flux	-	
V_{NMC}	Volume of the NMC domain	-	
ρ_{NMC}	NMC density	$3.8 \frac{g}{cm^3}$	
m_{NMC}	NMC mass fraction (70CAM, 80CAM)	0.7, 0.8	
SC_{NMC}	Specific capacity of NMC	$160 \frac{mAh}{g}$	
SA_{NMC}	Surface area of NMC in contact with LPSC	-	
R	Gas Constant	$8.314 \frac{J}{mol \cdot K}$	
T	Temperature	$293.15\ \mathrm{K}$	
F	Faraday's Constant	$96485 \frac{C}{mol}$	

2. Supplementary Figures

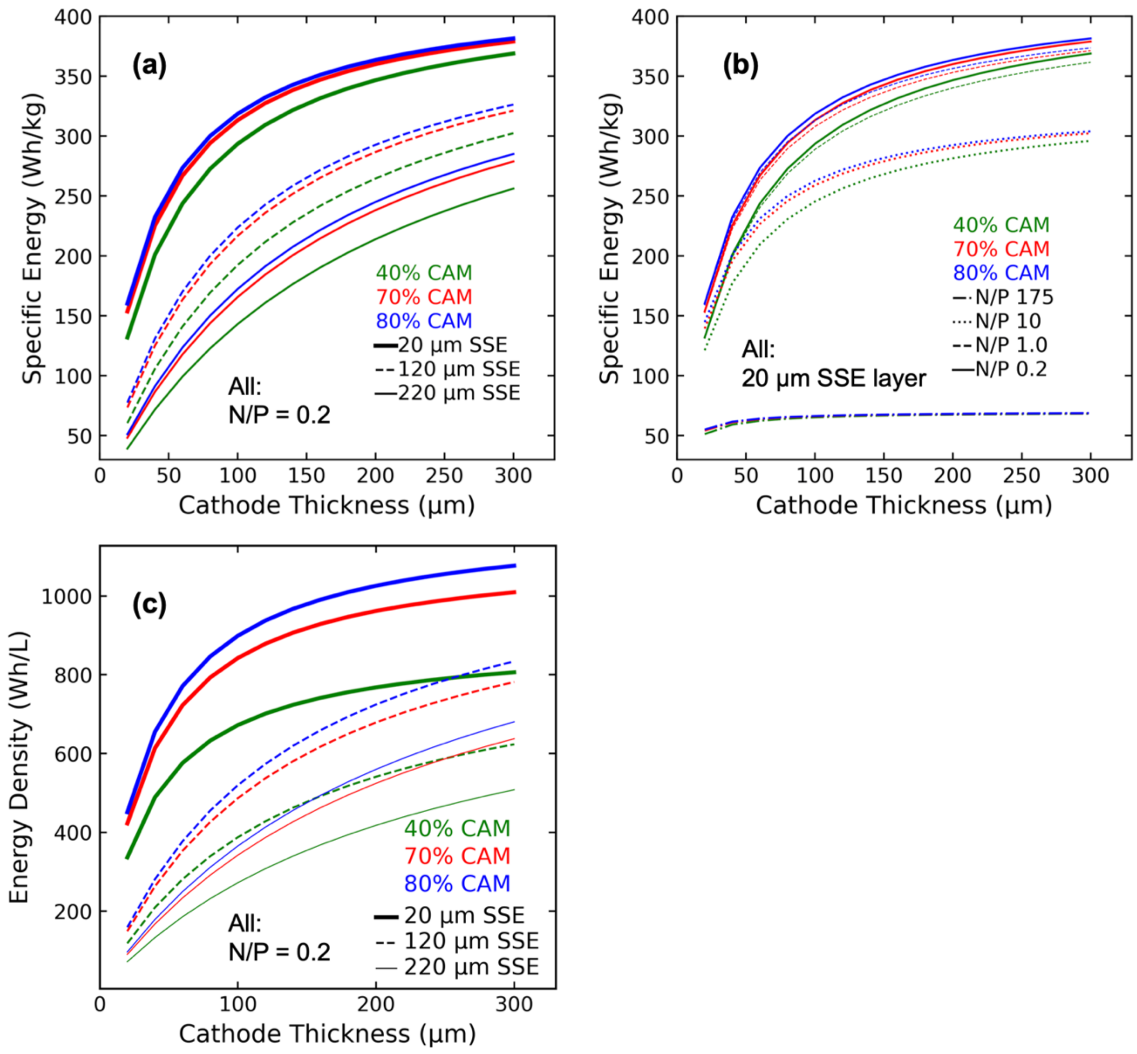

Figure S1. Specific energy and energy density calculations for ASLBs using NMC111 as the cathode active material (CAM). A Li metal foil anode is assumed for all cases. (a) Specific energy (Wh/kg) as a function of cathode thickness, mass% CAM in the cathode, and SSE electrolyte layer. N/P is constant at 0.2. (b) Specific energy (Wh/kg) as a function of cathode thickness, mass% CAM in the cathode, and N/P ratio. SSE layer thickness is constant at 20 μm. (c) Energy density (Wh/L) corresponding to the same conditions as panel a. All calculations used 3.8 V as the nominal voltage for NMC111 vs. Li/Li^{+}. No binder or carbon was included in the composite cathode, and the cell housing and terminals are ignored, so calculations represent a "best case" scenario.

The message of the calculations is that there is only one case in which a thicker composite cathode is not of high importance to achieve better energy density: that is the high N/P ratio of 175 (panel b), corresponding to use of a thick Li foil as the anode. When a thin foil or anode-free design are used, thickness of the composite cathode becomes a critical variable, and in all cases achieving cathode thickness > 100 μm impacts energy density considerably. Solving the problem of using a thin anode and thick cathode is of primary importance for practicality of all solid Li batteries (ASLBs).

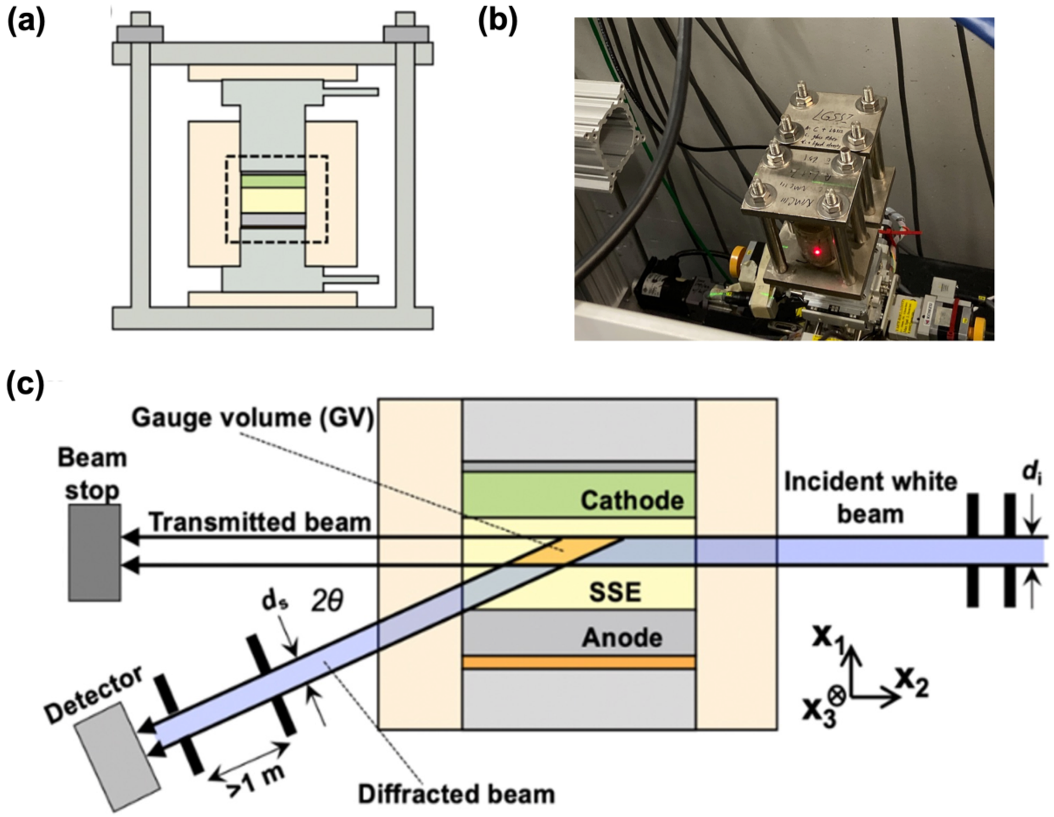

Figure S2. Energy dispersive X-ray diffraction (EDXRD) of sulfide solid electrolyte batteries with NMC111-LPSC composite cathodes. (a) Schematic of the batteries within the compression jig used for cycling. (b) Picture of two batteries in the experimental hutch of beamline 6BM-A at APS. (c) Concept of EDXRD, using an incident white beam of photons 20-200 keV. This beam penetrates the cell, and diffracted photons are detected at a constant angle of *2θ*. Diffraction data is from a well-defined gauge volume. By moving the cell, diffraction data can be obtained from different locations in the cell, enabling one to "slice" the cell components into different layers.

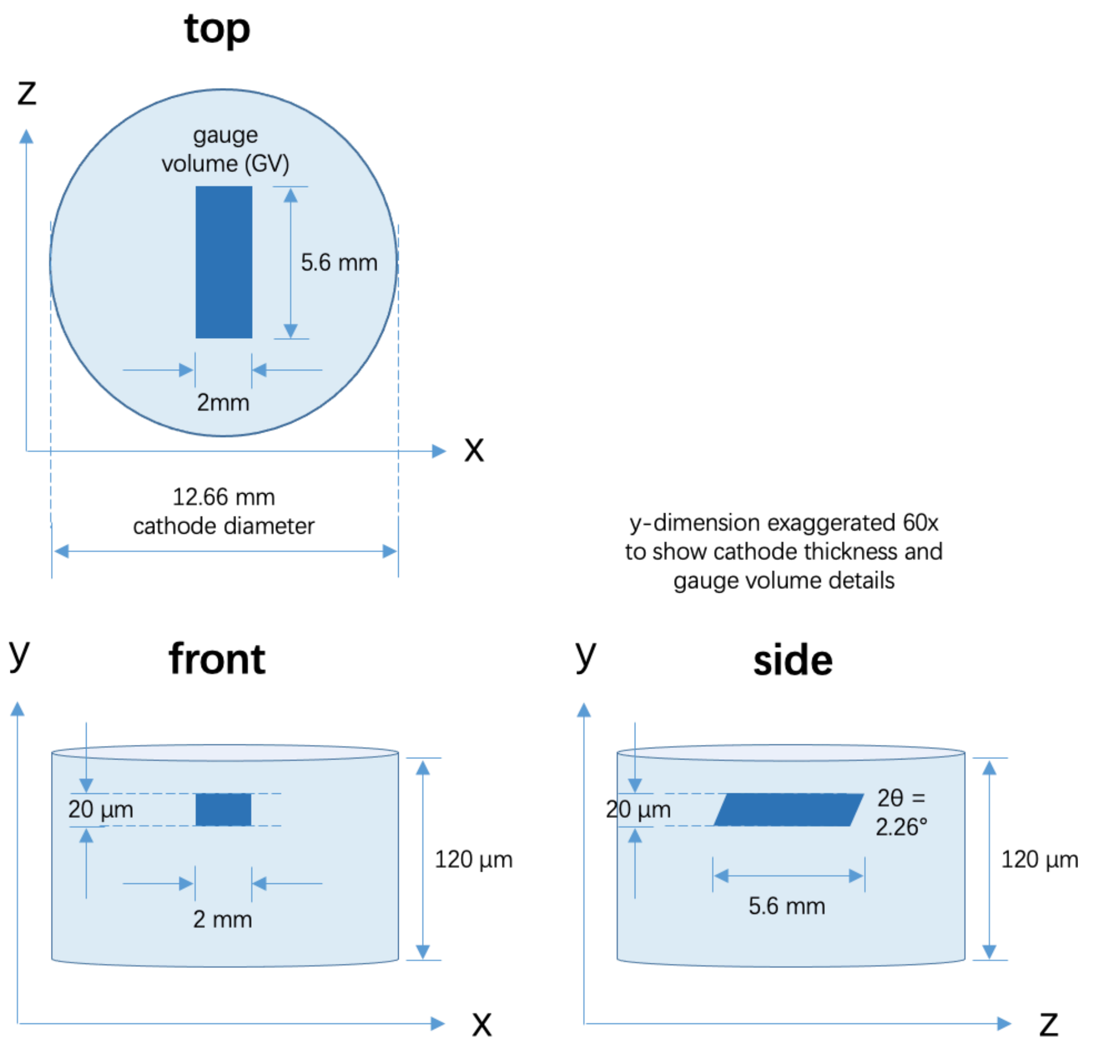

Figure S3. EDXRD gauge volume size shown to scale with the composite cathodes. The gauge volume had dimensions of 2 mm by ~5.6 mm by 0.02 mm defined by collimation slits of d_i = 0.02 mm and d_s = 0.2 mm. The y-dimension has been exaggerated 60x for the comparison of the vertical gauge volume and the cathode thickness. In these experiments, the gauge volume was 1.48% of the cathode volume.

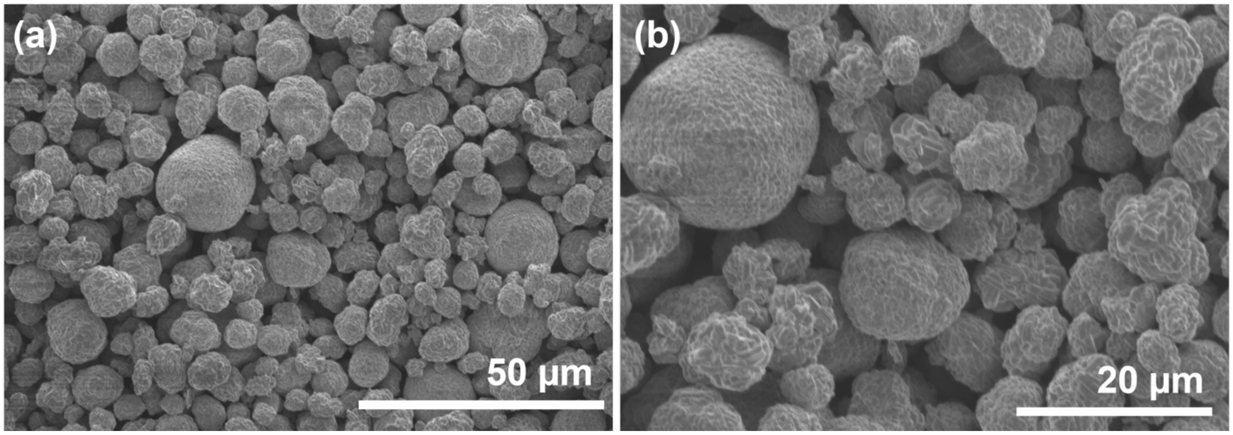

Figure S4. Scanning electron microscopy images of the NMC111 used in this study under different magnifications. The average size was 9.3 µm.

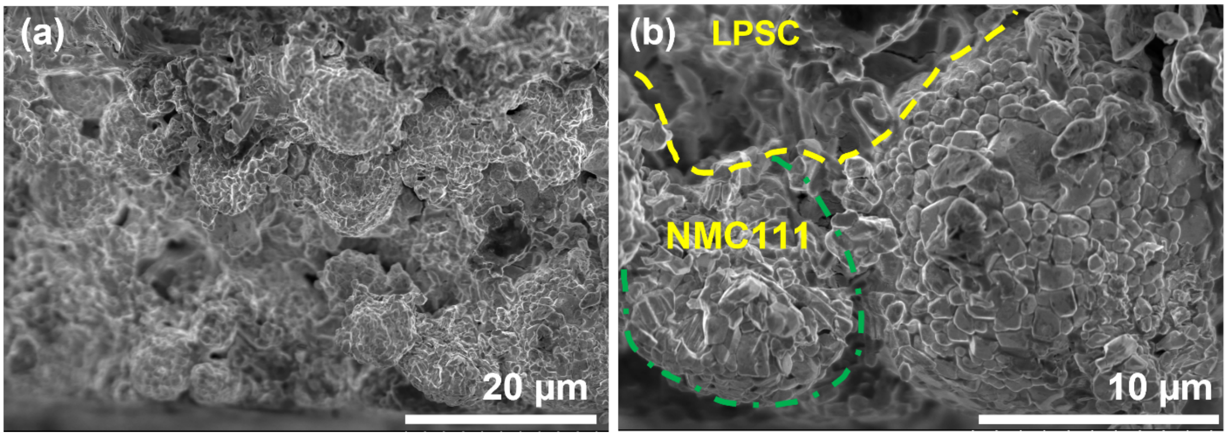

Figure S5. Scanning electron microscopy images of the mixture of NMC111 and LPSC.

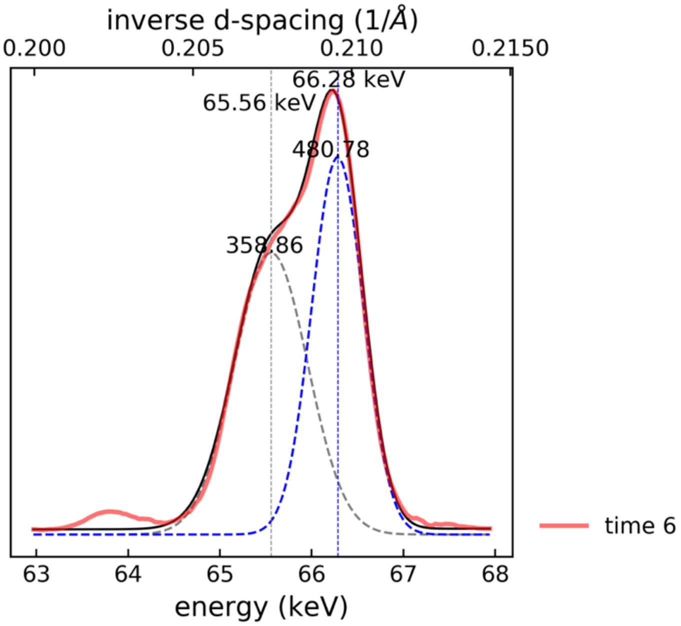

Figure S6. Demonstration of the method used to fit bifurcated NMC peaks. The NMC111 (003) is shown as an example. Red data is location 48 (EDXRD slice 3) in the middle of the 70% CAM cathode at time 6 (2.7 hours). It shows bifurcation, which was described by a double gaussian fit, shown in black. The left and right fitted peaks are shown in gray and blue dashed lines. Peak maxima are given in photon energy (keV). Peak heights are given in detector counts. The small peak at 63.8 keV is LPSC electrolyte in the composite cathode.

The weighted average of photon energy (and therefore d-spacing) was calculated according to the following formula. Gaussian peaks all have the same area, so height of the peaks was used for weighting, as in **Eq S14**.

Peak = energy1*(height1/(height1+ height2)) + energy2*(height2/(height1+ height2)) [S14]

A similar process was used in Li, et al. *Chem. Mater.* 2020, 32, 15, 6358–6364, where the parameters were calculated using a weighted average.[14]

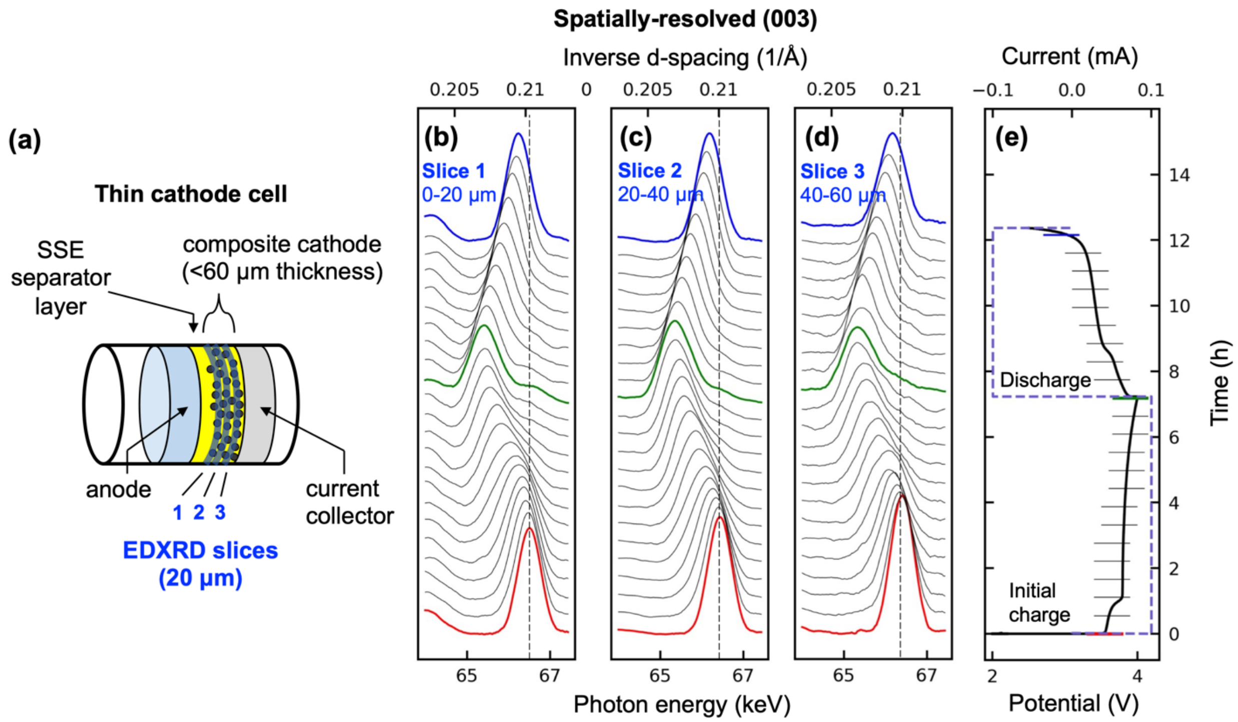

Figure S7. (a) The concept of an ASLB with a ~50 μm cathode, with EDXRD data taken in three 20 μm slices to observe non-uniformity through the cathode thickness. (b-e) Operando EDXRD for the 50 μm cathode cell at C/10, showing spatially-resolved measurement of the (003) reflection. Composition was 70% CAM with a coating. (b) Slice 1, depth of 0-20 μm. (c) Slice 2, depth of 20-40 μm. (d) Slice 3, depth of 40-60 μm. Slice 1 was only partially in the composite cathode, and a prominent LPSC peak can also be seen at ~64 keV. (e) Voltage profile during operando cycling.

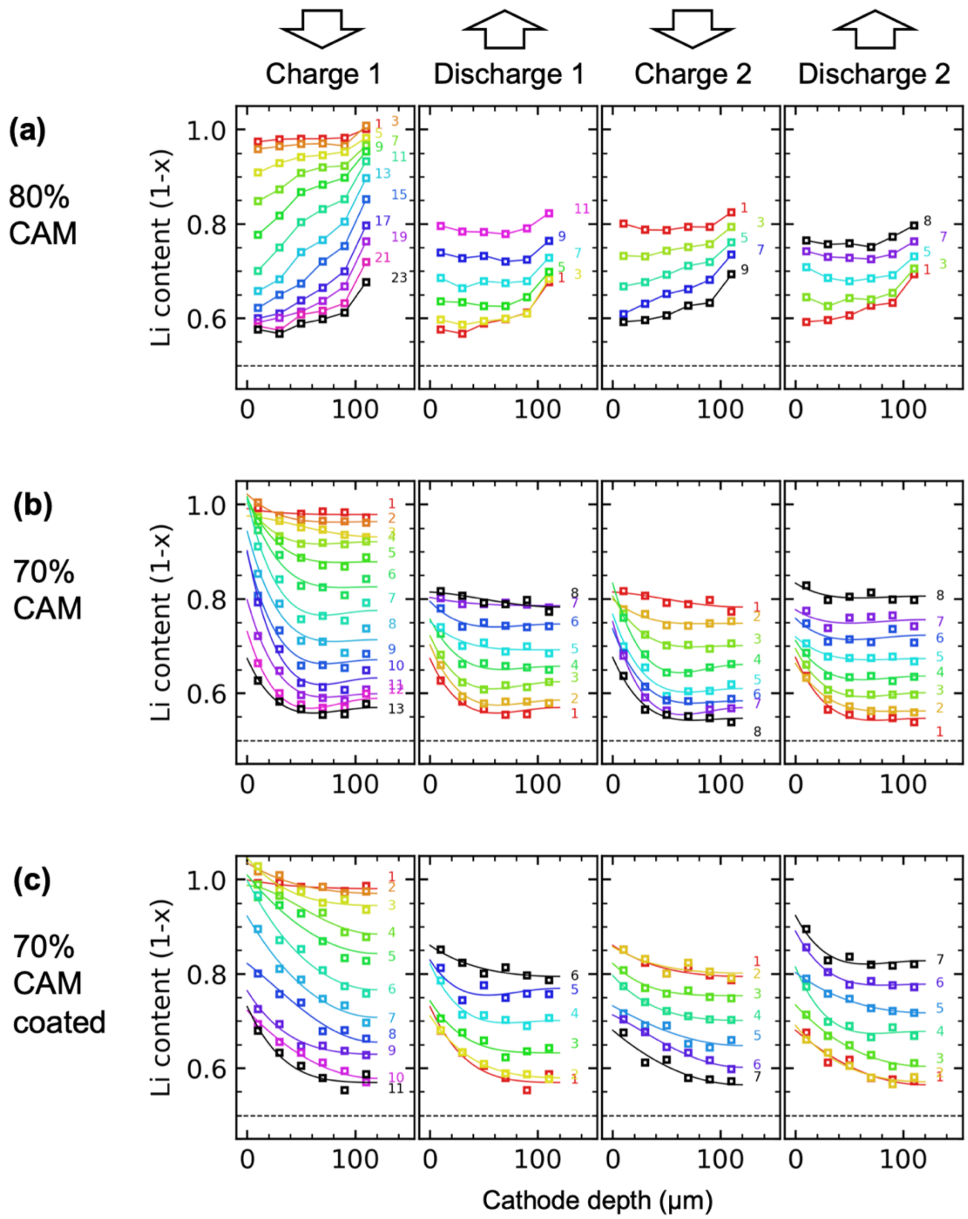

Figure S8. Lithiation profiles (as $(1-x)$ data) from operando EDXRD. During each cycling stage, colors follow the rainbow from initial time (red) to final time (black). (a) 80% CAM cell; (b) 70% CAM cell; (c) 70% CAM cell with LLSTO-coated NMC. For the 70% cells, the markers are EDXRD data while the lines are Legendre polynomial fits. For the 80% CAM cell, the markers are EDXRD data, with straight lines included to guide the eye.

The $(1-x)$ data in **Fig S8** are shown plotted as time-dependent Li^+ profiles through the cathode thickness. The sequential times during charge 1 are labeled, with each plotted as a different color on the spectrum from red to black. This is also shown for the eight times in the first discharge, second charge, and second discharge. For characterization of the Li^+ gradients in the 70% CAM cells, the cathode thickness was normalized to a reduced depth ξ and fit to a sum of orthogonal polynomials of increasing order, of the form in **Eq S15**.

$$c(\xi, t) \approx u_0(t)P_0(\xi) + u_1(t)P_2(\xi) + u_2(t)P_4(\xi) + \ldots \quad \text{[S15]}$$

The $P_{2n}(\xi)$ terms were even-order Legendre polynomials, which satisfy the requirement that the Li^+ concentration profile have a zero-flux boundary condition at the current collector. This method has been reported previously for both graphite and NMC532 electrodes in liquid-electrolyte cells.[15, 16] The resulting continuous profiles, using terms up to $P_4(\xi)$, are plotted as solid lines. The 80% cell lithiation profiles could not be fit with Legendre polynomials, even by including higher-order terms. This was because there was a discontinuity between the 80-100 μm and 100-120 μm slices, as seen in **Fig S8a**. This discontinuity was clear during all stages of cycling.

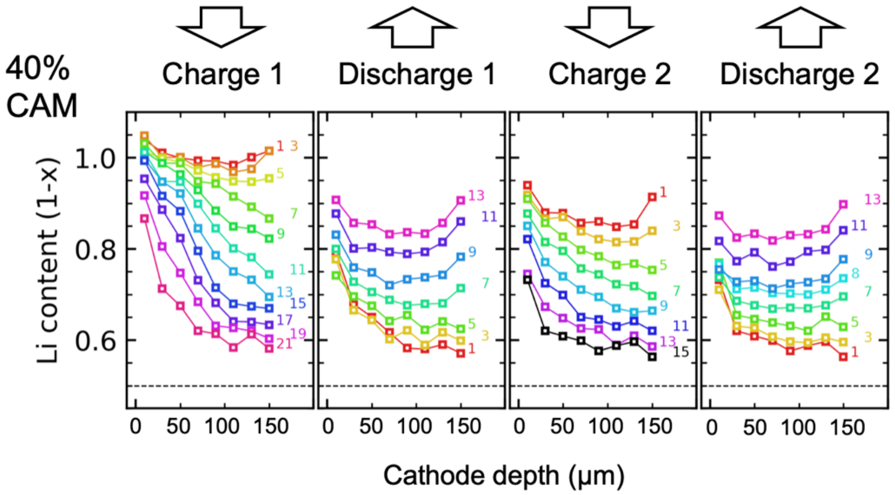

Figure S9. Lithiation profiles from EDXRD data for the 40% CAM cell. All conditions are as in **Fig S8**, except the 40% CAM cathode had a greater thickness.

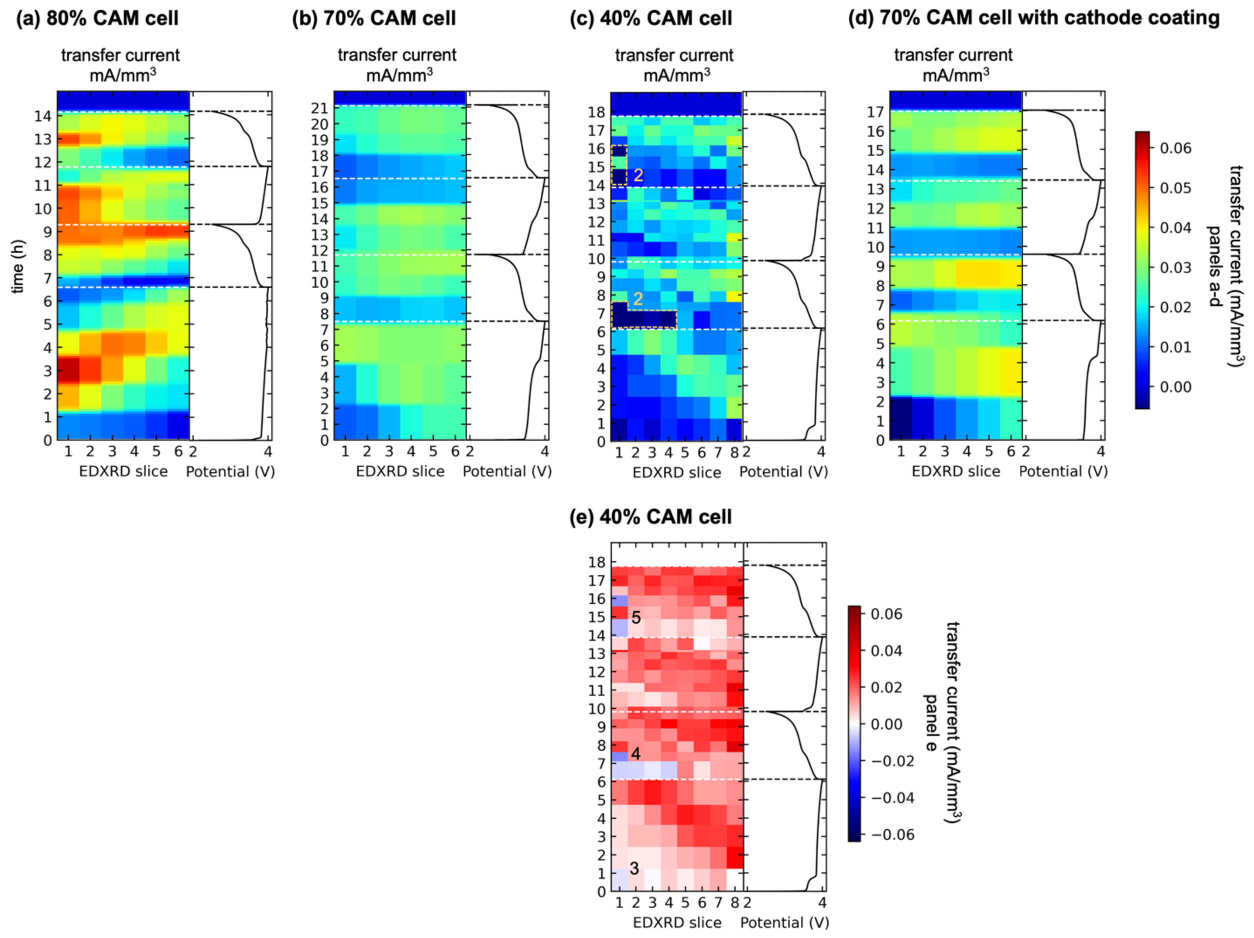

Figure S10. Local reaction rate or volumetric transfer current for initial cycling of NMC111-LPSC cathodes as a function of cathode composition. Cycling was at C/10. (a) 80% cathode active material (CAM); (b) 70% CAM; (c) 40% CAM; (d) 70% CAM with LLSTO-coated NMC. For each cell, local transfer current (in mA/mm^3) is shown correlated to the voltage profile. (e) 40% CAM shown with a diverging colormap to emphasize current reversal (in blue). EDXRD slice 1 was near the separator, while the highest slice was near the current collector.

In this study, cathode total mass was held constant at 30 mg and CAM mass% was varied. As all cycling was at C/10, this means the 80% CAM material experienced the highest current and 40% CAM the lowest. This was apparent in the results above, as expected. However, the times and locations of the most vigorous reaction in each cell varied significantly. During charge 1, a reaction wave passed across the 80% CAM cell, beginning at the separator at 2-3 hours, moving through the cathode interior, and being a maximum at the current collector at 5-6.5 hours. In the 70% and 40% CAM cells, this phenomenon was opposite, with the reaction wave beginning at the current collector and moving toward the separator as charge 1 progressed. Subsequent discharge and charge showed similar results, although the times were more brief.

The regions shown by note 2 in panel (c) revealed current reversal in the 40% CAM cell. To emphasize this, panel (e) shows the same data with a diverging colormap, where white is zero transfer current, and any current reversal (negative of the expected transfer current) is in blue. Note 3 shows an initial increase in `(1-x)` of 0.01, which may have been within the range of experimental error. Note 4 in discharge 1 shows a change in `(1-x)` in EDXRD slice 1 from 0.79 to 0.74, which was a significant current reversal. Note 5 in discharge 2 shows a change from 0.73 to 0.71, then a brief rise to 0.76, then a fall to 0.73. After that this `(1-x)` in this slice increased through the rest of discharge 2, conforming to what was occurring elsewhere in the cathode.

It was clear from the operando EDXRD data in **Fig S10** that there was also a lateral current distribution, because the beginnings of each cycling stage (e.g. charge 1, discharge 1, etc.) had below-average transfer currents in the gauge volume (with the exception of charge 2 in the 80% and 70% CAM cells). This means that immediately following a current switch, locations at the circumference of the cathode reacted first, and the center "caught up" within about an hour. However, this did not greatly affect the observed current distributions *through* the cathode thicknesses, which showed the strong dependence on CAM mass% that is the subject of this work (e.g. the 70%-80% flip). For this reason, this work did not focus on the lateral directions.

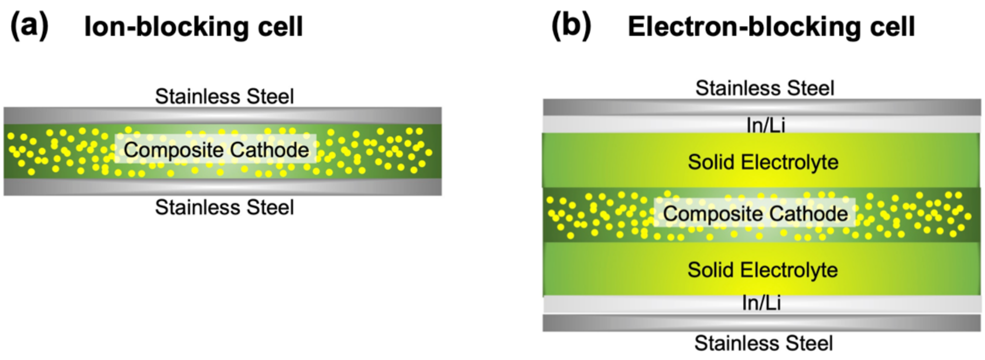

Figure S11. Schematic of blocking cells used for EIS testing. (a) Ion-blocking cell, which measures electronic conductivity. (b) Electron-blocking cell, which measures ionic conductivity.

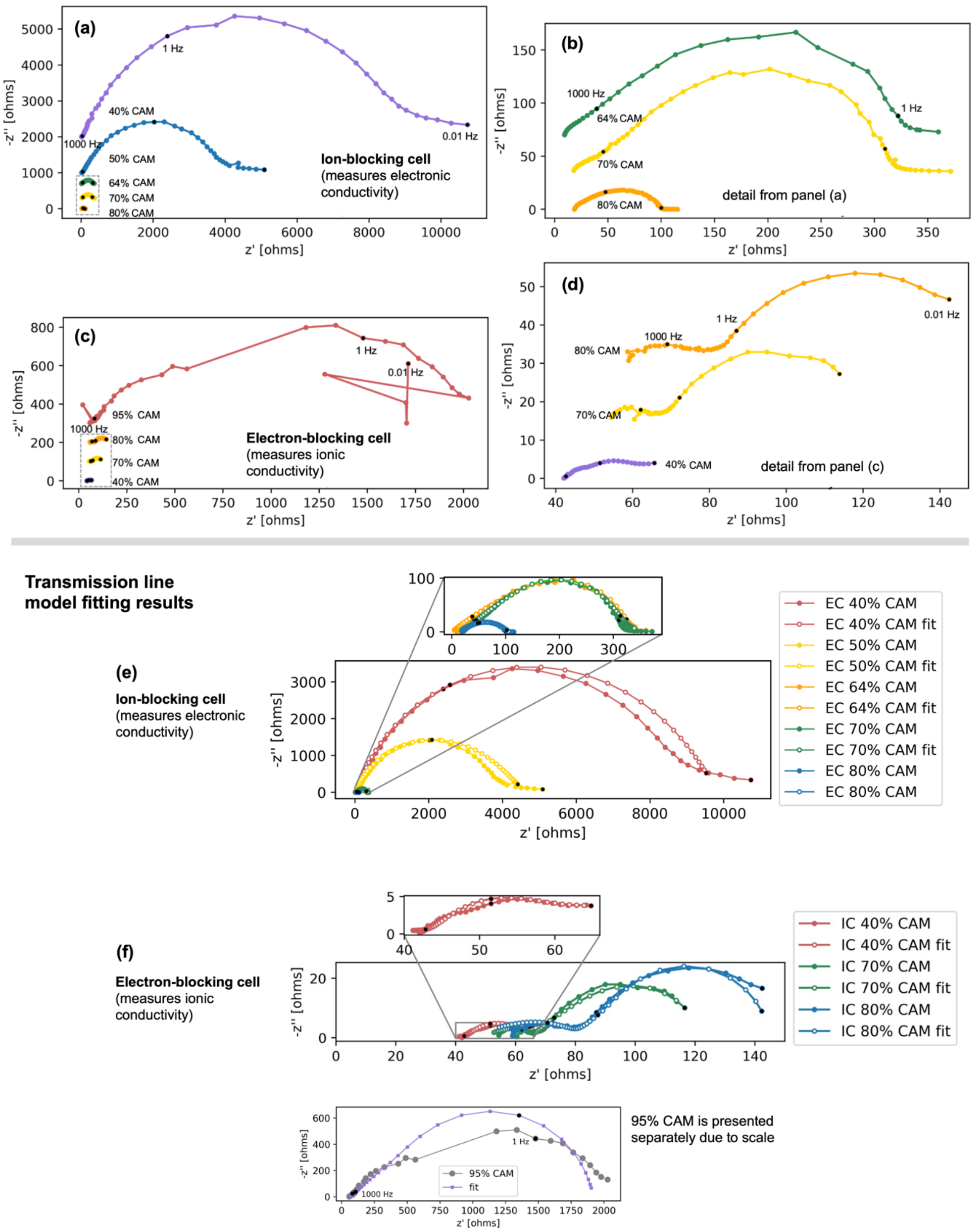

Figure S12. EIS of symmetric blocking cells. **(a-d)** Raw EIS results from the blocking cells in **Fig S11**, presented as Nyquist plots. The Nyquist plots are given an arbitrary y-axis offset to present multiple datasets on the same plot. Data at 1000, 1, and 0.01 Hz are emphasized. CAM percent is as indicated. (a) Ion-blocking cell, used to calculate electronic conductivity. (b) Detail from panel a. (c) Electron-blocking cell, used to calculate ionic conductivity. (d) Detail from panel c. **(e-f)** Model fitting results with open circles showing the fits. (e) Electron conducting/ion blocking. (f) Ion conducting/electron blocking.

Electronic and ionic conducting experiments were performed on all-solid-state battery (ASSB) cathodes. The amount of cathode active material (CAM) was varied relative to the amount of solid-state-electrolyte (SSE) present. All composite cathodes were 30 mg by mass with varying percentages of cathode active material. For the electron conducting/ion blocking experiments, the composite cathode was sandwiched between two pieces of stainless steel. For the ion conducting/electron blocking experiments, the composite cathode had 150 mg of SSE on each side as well as a 50 mg piece of In foil and a 1 mg piece of Li foil, with the indium against the SSE and the Li on the stainless steel.

Electrochemical impedance spectroscopy (EIS) was performed from 1 MHz to 0.01 Hz at a 10mV amplitude with 6 points per decade on a Biologic SP-150. Data from cells with an ion-blocking configuration are shown in **Fig S12a-b**. The Nyquist plots appeared as single depressed semicircles, some of which had a slight horizontal tail in the low frequency region. High frequency data contacted the x-axis near 0 Ω in all cases, and the compositions of the cathodes did not significantly change this part of the data. The impedance values in the low frequency region indicated the resistance to electronic conductivity across the composite cathodes. This resistance increased as the NMC111 percentage fell. At 80% NMC111, it was 110 Ω. This was because interconnected NMC111 particles provided a good conduction path across the cathode. However, when the NMC111 percentage fell to 50%, this value was 5000 Ω, and 40% was over 10,000 Ω. As the electronically insulating LPSC became a larger volume of the composite cathode, viable conduction paths through NMC111 particles were increasingly rare.

EIS data for cells with an electron-blocking configuration are shown in **Fig S12c-d**. These data revealed the ionic conductivity across the composite cathodes. In contrast to the ion-blocking data, these Nyquist plots showed that the cathode compositions affected both the x-axis crossing in the high frequency region and the overall width of the curve. This was because Li-ion conduction through the composite cathode limited the system. In general, an increase in the NMC111 percentage of the composite cathode increased the high frequency resistance and increased the width of the curve. This was because as the LPSC became a smaller overall volume fraction of the cathode, Li-ion conduction paths were more tortuous.

The model fitting results for electron conducting/ion blocking experiments and ion conducting/electron blocking experiments are shown in **Figs S12e and S12f** respectively.

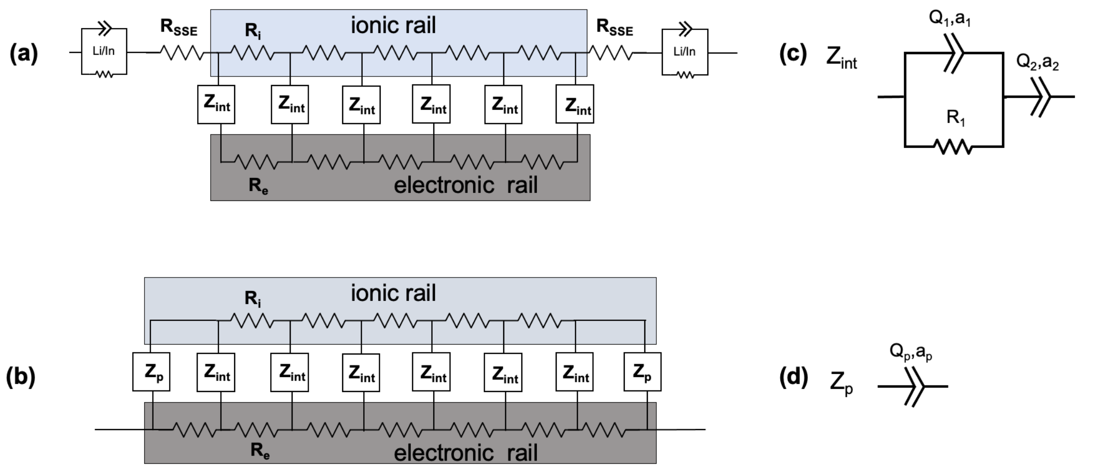

Figure S13. Transmission line model (TLM) of: (a) ion conducting/electron blocking experiments; and (b) electronic conducting/ion blocking experiments. (c) Z_{int} for all experiments. (d) Z_p end elements for electron conducting/ion blocking experiments.

Transmission line models (TLMs)

For the ionic conducting/electron blocking data a T-type open-open TLM was used (**Fig S13a**), calculated with **Eq S16**. For the electronic conducting/ion blocking data a symmetric T-type PP TLM was used (**Fig S13b**), calculated by **Eq S17**. The interfacial impedance Z_{int} describes the electrochemical reaction (**Fig S13c**). The end element impedance Z_p (**Fig S13d**) describes the connection between the SSE and the metal current collectors. In both equations Z_{el} and R_{ion} have units of ohm/m and Z_{int} has units of ohm m.

$$Z_{CC}(\omega) = \frac{R_{\mathrm{el}}R_{\mathrm{ion}}}{R_{\mathrm{el}}+R_{\mathrm{ion}}}\mathrm{L} + \frac{2\,{R_{\mathrm{ion}}}^{2}\sqrt{z_{\mathrm{int}}}}{(R_{\mathrm{el}}+R_{\mathrm{ion}})^{\frac{3}{2}}}\cdot\frac{\cosh\left(L\sqrt{\frac{R_{\mathrm{el}}+R_{\mathrm{ion}}}{Z_{\mathrm{int}}}}-1\right)}{\sinh\left(L\sqrt{\frac{R_{\mathrm{el}}+R_{\mathrm{ion}}}{z_{\mathrm{int}}}}\right)} \qquad \text{[S16]}$$

$$Z_{CC}(\omega) = \frac{R_{\mathrm{ion}}R_{\mathrm{el}}}{R_{\mathrm{ion}}+R_{\mathrm{el}}}\mathrm{L} + \frac{2\,{R_{\mathrm{el}}}^{2}\sqrt{z_{\mathrm{int}}}}{(R_{\mathrm{ion}}+R_{\mathrm{el}})^{\frac{3}{2}}}\cdot\frac{\cosh\left(L\sqrt{\frac{R_{\mathrm{ion}}+R_{\mathrm{el}}}{Z_{\mathrm{int}}}}-1\right)+\frac{1}{Z_p}\sqrt{z_{\mathrm{int}(R_{\mathrm{ion}}+R_{\mathrm{el}})}}\sinh\left(L\sqrt{\frac{R_{\mathrm{ion}}+R_{\mathrm{el}}}{Z_{\mathrm{int}}}}\right)}{\left\{1+\frac{1}{{Z_p}^{2}}z_{\mathrm{int}(R_{\mathrm{ion}}+R_{\mathrm{el}}}\right\}\sinh\left(L\sqrt{\frac{R_{\mathrm{ion}}+R_{\mathrm{el}}}{z_{\mathrm{int}}}}\right)\frac{2}{Z_p}\sqrt{z_{\mathrm{int}(R_{\mathrm{ion}}+R_{\mathrm{el}})}}\cosh\left(L\sqrt{\frac{R_{\mathrm{ion}}+R_{\mathrm{el}}}{z_{\mathrm{int}}}}\right)} \qquad \text{[S17]}$$

Many different TML variations were attempted for each type of experiment (7 for the IC data, 13 for the EC data). The final model used was selected for goodness of fit in addition to simplicity. For example, more complex versions of Z_{int} were explored, but the version shown in **Fig S13c** produced comparable results and had a straightforward form. Using the definitions developed in Siroma, the models had the qualities shown in **Table S5** below.[17]

Table S5. Transmission line models (TLMs) used, in terms of elements in Siroma, et al.

**Model**	**Type**	**Zion**	**Zel**	**Zint**	**End**
**IC**	T-type open-open	R_{ion}	R_{el}	R1/Q1 + Q2	Fixed<br>ZHFR= R and<br>ZLiIn = Q/R

Model	Type	Zion	Zel	Zint	Zp
**EC**	T-type PP	R_{ion}	R_{el}	R1/Q1	Qp

The model fitting results for both electron conducting/ion blocking experiments and ion conducting/electron blocking experiments are shown in **Fig S12e-f**. The fitted parameters are shown below for the ionic conductivity data (**Table S6**) and the electronic conductivity data (**Table S7**). It should be noted that the TLM results are relatively insensitive to some parameters in the models. For example, the ionic resistance (R_{ion}) has only a small impact on the electronic conductivity fits, as would be expected. The primary parameter determining the impedance in the ionic conductivity model is R_{ion}, and the primary parameter determining the impedance in the electronic conductivity model is R_{el}. For these models, the less-important resistance was allowed to vary freely during the fitting procedure. A global model to fit both experimental setups simultaneously is beyond the scope of this work. The approach used here has been used by other research groups in the field (Minnmann et al., 2021).[18]

Table S6. Fit parameters for T-type open-open ionic conductivity fits.

CAM mass%	R_{el} [ohm/cm]	R_{ion} [ohm/cm]	R1 [ohm cm]	Q1 [ohm cm]	a1	Q2 [ohm cm]	a2
40%	486.2186	5363.493	106.10121	0.128480	0.024549	0.020878	0.436231
70%	7187.556	9236.25	0.048526	2.96482	0.55130	0.022629	0.819895
80%	7270.534	11866.449	0.058496	1.701138	0.560498	0.0322893	0.774103
95%	6749.6147	211851.11	0.251652	0.0007087	0.13592	2.6695974	0.887930

Table S7. Fit parameters for T-type PP electronic conductivity fits.

CAM mass%	R_{ion} [ohm/cm]	R_{el} [ohm/cm]	R1 [ohm cm]	Q1 [ohm cm]	a1	Q_p [$ohm^{0.5}$]	a_p
40%	3678.82	1312045.2	23.816	42.87	0.370	2733.25	0.824
50%	373.02	396248	9079.04	19.53	0.2616	2420.99	0.789
64%	8.85	55208	174.76	6.712	0.8895	328.49	0.0285
70%	1666.43	32743	119.65	4.77	0.885	867.52	0.099
80%	1669.8	9828.37	0.97460374	98184.889	0.7135	1109.83	0.48937
70%-LLSTO	3255.92	9968	0.0085	2.472	0.7868	0.00065	0.043

The electronic and ionic conductivities for composite cathodes calculated from this data using the EIS models presented here are given in **Table S8** (and in **Fig 4a**). The resulting tortuosity factors are also given in **Table S8** (and in **Fig 4b**).

Table S8. Conductivity and tortuosity factor values from **Fig 4** in the main text.

CAM mass%	CAM vol%*	LPSC vol%*	Electronic conductivity (EC) S/cm	Tortuosity factor from EC	Ionic conductivity (IC) S/cm	Tortuosity factor from IC
100	86%	-	1.88×10^{-4}	-	-	-
95	78%	8%	-	-	3.785×10^{-6}	392.44
80	56%	30%	8.16×10^{-5}	1.28	6.758×10^{-5}	15.62
70 coated	44%	42%	2.66×10^{-5}	3.54	8.045×10^{-5}	11.85
70	44%	42%	2.45×10^{-5}	3.37	8.682×10^{-5}	9.63
64	38%	48%	1.45×10^{-5}	4.92	-	-
50	26%	60%	2.02×10^{-6}	24.52	-	-
40	20%	66%	6.11×10^{-7}	60.18	1.495×10^{-4}	2.49
0	0%	86%	-	-	1.900×10^{-3}	-

* calculated assuming 14% void[5]

Tortuosity factors for the NMC and LPSC phases were calculated three ways in this work. These are compiled in **Table S9**. (1) From individual symmetric blocking cell EIS experiments, shown in blue. (Also see **Table S8**.) (2) From power-law fits to all EIS experiments, deriving tortuosity factor as a function of volume fraction for NMC and LPSC, shown in green. (3) From the COMSOL model correlated to EDXRD data for the 70% CAM and 80% CAM cells, shown in yellow.

Table S9. All tortuosity factor values calculated in this work.

		**Method:**	From EIS		From fit of all EIS experiments		From fit with COMSOL model of 70% CAM and 80% CAM	
				a_i **values:**	2.511	2.365	2	2.7
**Phase:**	**NMC**	**LPSC**	**NMC**	**LPSC**	**NMC**	**LPSC**	**NMC**	**LPSC**
**CAM mass%**	**vol frac**	**vol frac**	τ^2_{NMC}	τ^2_{LPSC}	τ^2_{NMC}	τ^2_{LPSC}	τ^2_{NMC}	τ^2_{LPSC}
95	0.78	0.08	-	392.44	1.87	392.82	1.64	915.50
80	0.56	0.3	1.28	15.62	4.29	17.24	3.19	25.81
70	0.44	0.42	3.37	9.63	7.86	7.78	5.17	10.40
64	0.38	0.48	4.92	-	11.35	5.67	6.93	7.26
50	0.26	0.6	24.52	-	29.44	3.35	14.79	3.97
40	0.2	0.66	60.18	2.49	56.90	2.67	25.00	3.07

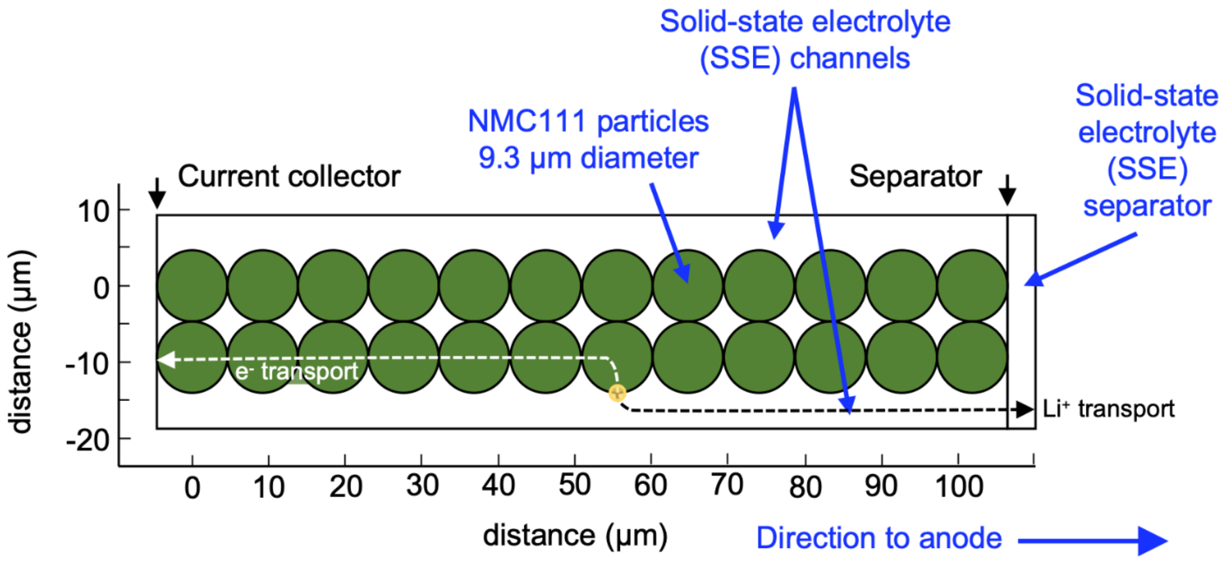

Figure S14. Domain used for COMSOL modeling of the composite cathodes. CAM particles shown in green. Two rows of these represent the active material in the composite electrode. LPSC is modeled as a continuous single-ion conducting phase, show in in white. The current collector is on the left side of the domain, and the SSE separator is on the right. The SSE separator is assigned the bulk conductivity of LPSC $\sigma_{LPSC,\mathrm{bulk}}$. The two SSE channels across the composite cathode are assigned the effective conductivity of LPSC, $\sigma_{LPSC,\mathrm{eff}}$, calculated from the LPSC phase fraction ϵ_{LPSC} and tortuosity factor τ^2_{LPSC}. The CAM particles are assigned the effective conductivity of NMC111 at the state of charge (SOC) of the local material, $\sigma_{NMC,\mathrm{eff}}$, calculated from **Eq S5**, the NMC phase fraction ϵ_{NMC} and tortuosity factor τ^2_{NMC}. The dashed lines show a hypothetical transport path for electrons and Li ions after a charging reaction at the spot marked by an orange circle.

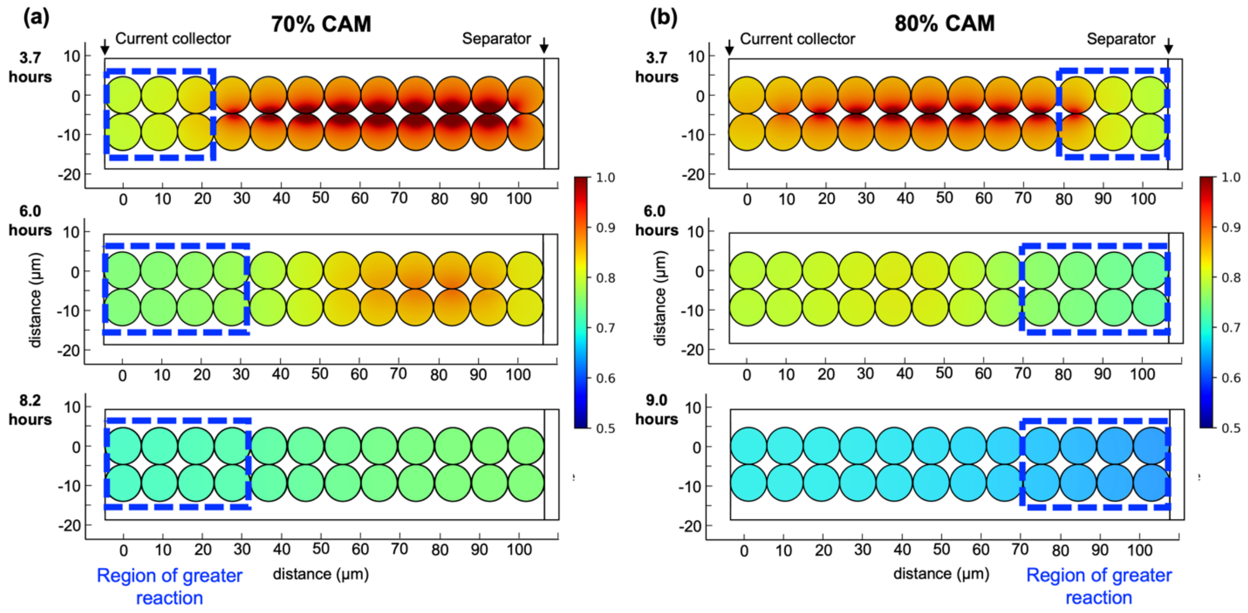

Figure S15. COMSOL simulations performed as those in **Fig 5** of the main text, with the i_o expression used from Liu et al., ref 8, instead of the expression used in the main text from Park et al., ref 12. (a) COMSOL simulation of initial charge of a 70% CAM cell. (b) COMSOL simulation of initial charge of an 80% CAM cell.

Results were similar regardless of the form of i_o chosen, suggesting that the kinetic expression did not have a large impact on the lithiation gradients. These gradients were primarily a consequence of the effective conductivities across the composite cathode thickness, and therefore the phase fractions and tortuosity factors.

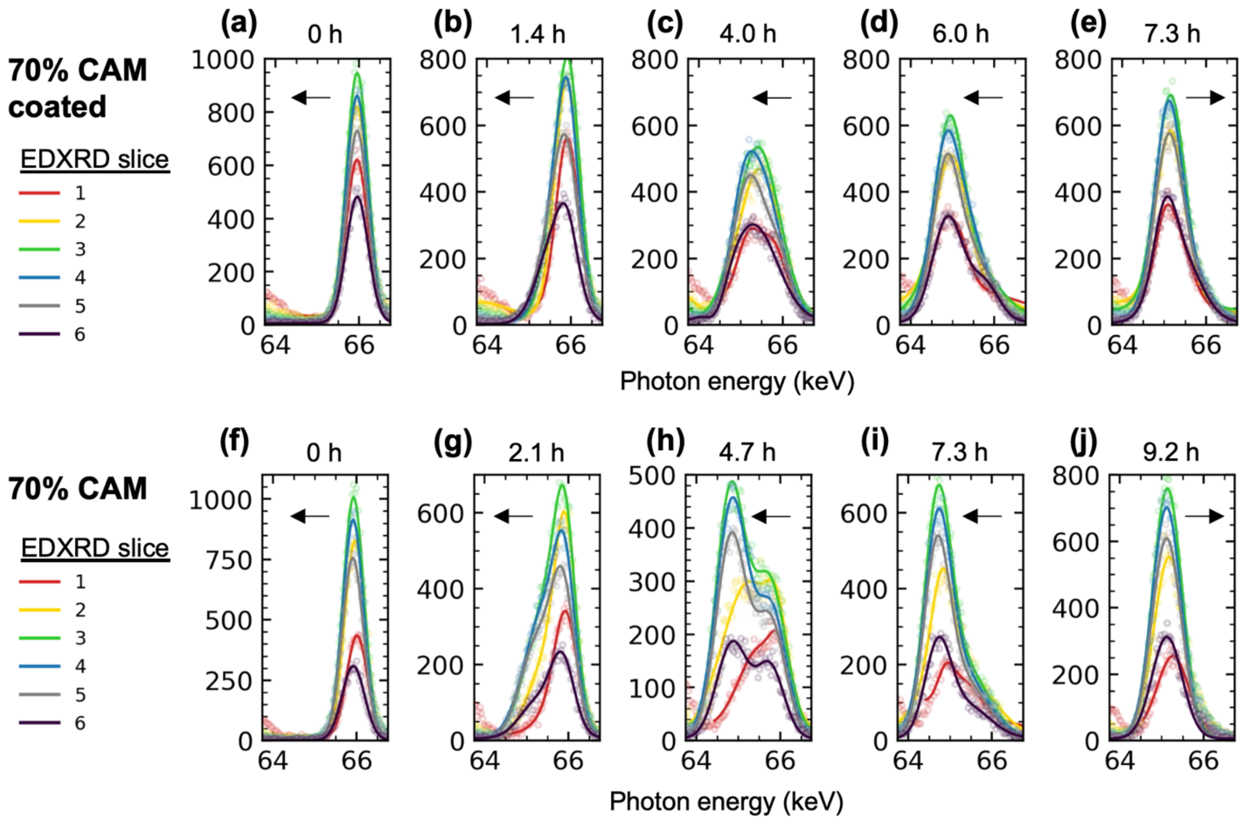

Figure S16. Operando evolution of the NMC111 (003) reflection during charge 1, as a function of time and depth in the cathode. (a-e) 70% CAM with a coating. (f-j) uncoated 70% CAM. Data were collected dynamically while under load, and the arrows show the direction the peaks were moving in during the experiment.

In each figure, the NMC111 (003) peak is shown at four times during charge 1 and at one time at the beginning of discharge 1. Markers show the raw EDXRD data, and the solid lines show a double Gaussian fit resolving the data into two distinct peaks. Depth through the cathode is shown by color, as EDXRD slices 1-6 go from red to purple. The times of these data are marked by dashed lines in **Fig 6** of the main text. The conclusion is that peak bifurcation was more significant in the uncoated cell.

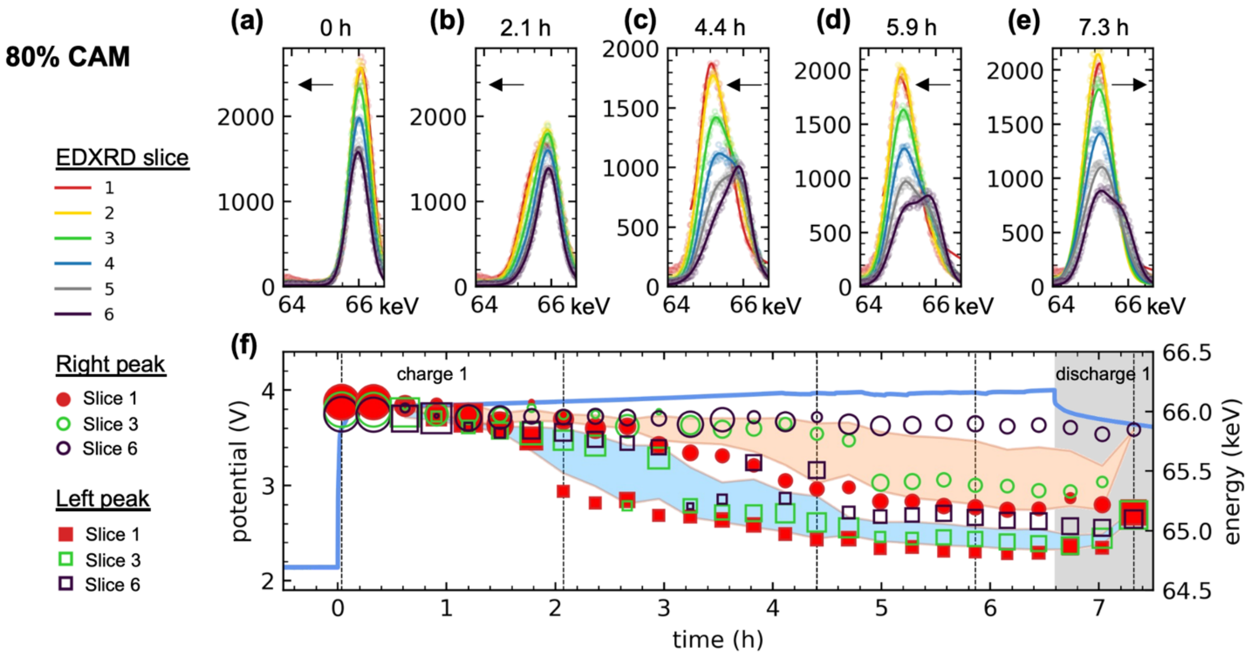

Figure S17. Operando evolution of the NMC111 (003) reflection during charge 1, as a function of time and depth in the cathode for the 80% CAM cell. (a-e) A zoom of the peak is shown at five times, with raw data as transparent markers and a double Gaussian fit as a solid line: (a) 0 h; (b) 2.1 h; (c) 4.4 h; (d) 5.9 h; (e) 7.3 h, which was during discharge 1. Depth in the cathode is indicated by color. (f) On the voltage profile, the photon energies of the double peaks are shown, with the right and left peaks given by circles and squares. Peak magnitude is indicated by the marker size. Color-shaded regions show the weighted standard deviations of the peak photon energies.

The marker for slice 1 is emphasized in this figure as solid red. The peak at this location showed only small peak bifurcation, and only from 2.1 hours to about 3.7 hours. In contrast, the peak at slice 6 remained bifurcated throughout charge 1 and remained bifurcated well into discharge 1.

Supplemental References

1. Cao, D.; Zhang, Y.; Nolan, A. M.; Sun, X.; Liu, C.; Sheng, J.; Mo, Y.; Wang, Y.; Zhu, H., "Stable thiophosphate-based all-solid-state lithium batteries through conformally interfacial nanocoating." *Nano Letters,* **2019,** *20* (3), 1483-1490.

2. Marschilok, A. C.; Bruck, A. M.; Abraham, A.; Stackhouse, C. A.; Takeuchi, K. J.; Takeuchi, E. S.; Croft, M.; Gallaway, J. W., "Energy dispersive X-ray diffraction (EDXRD) for operando materials characterization within batteries." *Physical Chemistry Chemical Physics,* **2020,** *22* (37), 20972-20989.

3. Waseda, Y.; Matsubara, E.; Shinoda, K., *X-ray diffraction crystallography: introduction, examples and solved problems*. Springer Science & Business Media: 2011.

4. Buchberger, I.; Seidlmayer, S.; Pokharel, A.; Piana, M.; Hattendorff, J.; Kudejova, P.; Gilles, R.; Gasteiger, H. A., "Aging analysis of graphite/$LiNi_{1/3}Mn_{1/3}Co_{1/3}O_2$ cells using XRD, PGAA, and AC impedance." *Journal of the Electrochemical Society,* **2015,** *162* (14), A2737.

5. Bielefeld, A.; Weber, D. A.; Janek, J. r., "Modeling effective ionic conductivity and binder influence in composite cathodes for all-solid-state batteries." *ACS Applied Materials & Interfaces,* **2020,** *12* (11), 12821-12833.

6. Landesfeind, J.; Hattendorff, J.; Ehrl, A.; Wall, W. A.; Gasteiger, H. A., "Tortuosity determination of battery electrodes and separators by impedance spectroscopy." *Journal of the Electrochemical Society,* **2016,** *163* (7), A1373.

7. Sharma, N.; Vasconcelos, L. S. d.; Hassan, S.; Zhao, K., "Asynchronous-to-synchronous transition of Li reactions in solid-solution cathodes." *Nano Letters,* **2022,** *22* (14), 5883-5890.

8. Liu, C.; Lombardo, T.; Xu, J.; Ngandjong, A. C.; Franco, A. A., "An Experimentally-Validated 3D Electrochemical Model Revealing Electrode Manufacturing Parameters' Effects on Battery Performance." *Energy Storage Materials*, **2023**, 54, 156–163.

9. Crawford, A. J.; Choi, D.; Balducci, P. J.; Subramanian, V. R.; Viswanathan, V. V., "Lithium-ion battery physics and statistics-based state of health model." *Journal of Power Sources,* **2021,** *501*, 230032.

10. Rucci, A.; Ngandjong, A. C.; Primo, E. N.; Maiza, M.; Franco, A. A., "Tracking variabilities in the simulation of Lithium Ion Battery electrode fabrication and its impact on electrochemical performance." *Electrochimica Acta,* **2019,** *312*, 168-178.

11. Liu, P.; Xu, R.; Liu, Y.; Lin, F.; Zhao, K., "Computational modeling of heterogeneity of stress, charge, and cyclic damage in composite electrodes of Li-ion batteries." *Journal of the Electrochemical Society,* **2020,** *167* (4), 040527.

12. Park, J.; Zhao, H.; Kang, S. D.; Lim, K.; Chen, C.-C.; Yu, Y.-S.; Braatz, R. D.; Shapiro, D. A.; Hong, J.; Toney, M. F., "Fictitious phase separation in Li layered oxides driven by electro-autocatalysis." *Nature Materials,* **2021,** *20* (7), 991-999.

13. Ansah, S.; Hyun, H.; Shin, N.; Lee, J.-S.; Lim, J.; Cho, H.-H., "A modeling approach to study the performance of Ni-rich layered oxide cathode for lithium-ion battery." *Computational Materials Science,* **2021,** *196*, 110559.

14. Li, Z.; Yin, L.; Mattei, G. S.; Cosby, M. R.; Lee, B.-S.; Wu, Z.; Bak, S.-M.; Chapman, K. W.; Yang, X.-Q.; Liu, P., "Synchrotron Operando Depth Profiling Studies of State-of-Charge Gradients in Thick Li ($Ni_{0.8}Mn_{0.1}Co_{0.1}$) O_2 Cathode Films." *Chemistry of Materials,* **2020,** *32* (15), 6358-6364.

15. Okasinski, J. S.; Shkrob, I. A.; Rodrigues, M.-T. F.; Raj, A.; Prado, A. Y.; Chuang, A. C.; Pidaparthy, S. S.; Abraham, D. P., "Time-Resolved X-ray Operando Observations of Lithiation Gradients across the Cathode Matrix and Individual Oxide Particles during Fast Cycling of a Li-Ion Cell." *Journal of the Electrochemical Society,* **2021,** *168* (11), 110555.

16. Okasinski, J. S.; Shkrob, I. A.; Chuang, A.; Rodrigues, M.-T. F.; Raj, A.; Dees, D. W.; Abraham, D. P., "In situ X-ray spatial profiling reveals uneven compression of electrode assemblies and steep lateral gradients in lithium-ion coin cells." *Physical Chemistry Chemical Physics,* **2020,** *22* (38), 21977-21987.

17. Siroma, Z.; Fujiwara, N.; Yamazaki, S.-i.; Asahi, M.; Nagai, T.; Ioroi, T., "Mathematical solutions of comprehensive variations of a transmission-line model of the theoretical impedance of porous electrodes." *Electrochimica Acta,* **2015,** *160*, 313-322.

18. Minnmann, P.; Quillman, L.; Burkhardt, S.; Richter, F. H.; Janek, J., "Editors' Choice—Quantifying the Impact of Charge Transport Bottlenecks in Composite Cathodes of All-Solid-State Batteries." *Journal of the Electrochemical Society,* **2021,** *168* (4), 040537.
